# Dissecting the Root Phenotypic and Genotypic Variability of the Iowa Mung Bean Diversity Panel

**DOI:** 10.3389/fpls.2021.808001

**Published:** 2022-01-27

**Authors:** Kevin O. Chiteri, Talukder Zaki Jubery, Somak Dutta, Baskar Ganapathysubramanian, Steven Cannon, Arti Singh

**Affiliations:** ^1^Department of Agronomy, Iowa State University, Ames, IA, United States; ^2^Department of Mechanical Engineering, Iowa State University, Ames, IA, United States; ^3^Department of Statistics, Iowa State University, Ames, IA, United States; ^4^USDA—Agricultural Research Service, Corn Insects and Crop Genetics Research Unit, Ames, IA, United States

**Keywords:** root system architecture, GWAS, high throughput phenotyping, phenomics, pulses

## Abstract

Mung bean [*Vigna radiata* (L.) Wilczek] is a drought-tolerant, short-duration crop, and a rich source of protein and other valuable minerals, vitamins, and antioxidants. The main objectives of this research were (1) to study the root traits related with the phenotypic and genetic diversity of 375 mung bean genotypes of the Iowa (IA) diversity panel and (2) to conduct genome-wide association studies of root-related traits using the Automated Root Image Analysis (ARIA) software. We collected over 9,000 digital images at three-time points (days 12, 15, and 18 after germination). A broad sense heritability for days 15 (0.22–0.73) and 18 (0.23–0.87) was higher than that for day 12 (0.24–0.51). We also reported root ideotype classification, i.e., PI425425 (India), PI425045 (Philippines), PI425551 (Korea), PI264686 (Philippines), and PI425085 (Sri Lanka) that emerged as the top five in the topsoil foraging category, while PI425594 (unknown origin), PI425599 (Thailand), PI425610 (Afghanistan), PI425485 (India), and AVMU0201 (Taiwan) were top five in the drought-tolerant and nutrient uptake “steep, cheap, and deep” ideotype. We identified promising genotypes that can help diversify the gene pool of mung bean breeding stocks and will be useful for further field testing. Using association studies, we identified markers showing significant associations with the lateral root angle (LRA) on chromosomes 2, 6, 7, and 11, length distribution (LED) on chromosome 8, and total root length-growth rate (TRL_GR), volume (VOL), and total dry weight (TDW) on chromosomes 3 and 5. We discussed genes that are potential candidates from these regions. We reported beta-galactosidase 3 associated with the LRA, which has previously been implicated in the adventitious root development *via* transcriptomic studies in mung bean. Results from this work on the phenotypic characterization, root-based ideotype categories, and significant molecular markers associated with important traits will be useful for the marker-assisted selection and mung bean improvement through breeding.

## Introduction

There is an increasing demand, particularly in Western cultures, for plant-based protein sources, including analogs of meat, egg, and dairy (Wild et al., 2014; Joshi and Kumar, 2016; Niva et al., 2017; Aschemann-Witzel et al., 2020). Numerous factors influence this change, including social, political, environmental, ethical, health-focused, technological, and economical (Vinnari, 2008; Markiewicz, 2010). Pulses such as lentils (*Lens culinaris*), horse beans (*Dolichos* spp.), lupins (*Lupinus albus* L.), common beans (*Phaseolus vulgaris*), chickpea (*Cicer arietinum*), field peas (*Pisum sativum*), cowpeas (*Vigna unguiculata*), fava beans (*Vicia faba*), mung bean [*Vigna radiata* (L.) Wilczek], urd beans (*Vigna mungo*), and food-grade soybeans (*Glycine max*) have consistently been used as protein sources in the global south (Niva et al., 2017). The plant protein demand has been fueled by the sustainable production of pulses coupled with their health benefits and the production of meat analogs (Wild et al., 2014; Niva et al., 2017). Residents of sub-Saharan Africa, the Caribbeans, and South America consume more than 10 kg/capita/year of pulses, compared to 3 kg/capita/year among Western cultures (Akibode and Maredia, 2012).

Mung bean (*V. radiata* L. Wilczek), initially domesticated in India, is now cultivated in over 7 million hectares worldwide (Nair et al., 2019; Aski et al., 2021). Mung bean is a short-duration crop, usually between 60 and 90 days from planting to harvest (Sandhu and Singh, 2021). Mung beans, being relatively heat- and drought-tolerant, may be helpful in the agricultural adaptation to climate change (Pataczek et al., 2018; Wang et al., 2018). Mung bean is easily digestible, with seed composition of 22–28% protein, 1–1.5% fat, and 60–65% carbohydrates, as well as minerals, vitamins, and antioxidants (Jahan et al., 2020; Aski et al., 2021; Sandhu and Singh, 2021). Mung beans are consumed as whole grain, sprouted gram, split dhal, and mung bean flour in Indian dishes (Nair et al., 2019; Aski et al., 2021). The complementation of mung beans with cereals provides a balanced intake of required nutrients. In Western cultures, mung beans are consumed mostly as sprouts and, more recently, as processed products such as meat substitutes, egg substitutes, chips, no-nut butter, and pasta (Sandhu and Singh, 2021). The demand for plant-based protein in the United States has led to enhancing existing and establishment of breeding programs at United States institutions, including at Iowa State University (Sandhu and Singh, 2021). However, due to limited breeding efforts in North America, there is a knowledge gap in agronomic trait diversity including root traits that are emerging as important area of research and breeding efforts (Lynch, 2007; White et al., 2013; Burridge et al., 2017).

Root system architecture (RSA) can be defined as the morphology of the root system at many scales, both global (i.e., the entire root system) and local (i.e., primary and lateral root levels), as well as the spatial variability of the morphology (Hodge et al., 2009; Rogers and Benfey, 2015; Lobet et al., 2019; Aski et al., 2021). The morphometric traits include the number, length, volume, mass, shape, angle, depth, etc. The spatio-temporal variation seen in RSA of different plants reflects the phenotypic plasticity and the genotype × environment interaction (Rogers and Benfey, 2015; Lobet et al., 2019). Roots have a great impact on yield and plant fitness by providing plants with the structural stability, nutrient foraging, plant-microbe interactions, preventing soil erosion, aeration, and water extraction (Hodge et al., 2009; Rogers and Benfey, 2015).

The desired root phenotypes by plant breeders will be ones that enhances plant adaptation to the edaphic stress while maintaining or increasing yields, for example, deeper and proliferating roots are desired during water-deficient stresses in the changing climate (Gaur et al., 2008; Aski et al., 2021). Lynch and Brown (2001) coined the term “topsoil foraging ideotype,” which is characterized by proliferation of lateral roots, long root hairs, association with mycorrhizal fungi, and suited to uptake of the immobile phosphorus mineral from the topsoil stratum (White et al., 2013). The “steep, cheap, and deep” ideotype (Lynch, 2013) optimizes on the uptake of water and the soluble nitrogen in the soil minimizing leaching. The “steep, cheap, deep” is characterized by thick and long primary roots, high affinity for N by epidermal cells, and the high concentration of cortical aerenchyma cells (White et al., 2013). Falk et al. (2020b) used the term “informative root” (iRoot) category to capture the biological significance of the captured root traits as would simulate field conditions. They reported that the topsoil foraging had a faster total root length-growth rate (TRL_GR), wider (WID), and a large TRL upper root ratio (TRL_Upper_). The steep, cheap, and deep ideotype contained a deeper primary root length (PRL), faster TRL_GR, steep lateral root angles (LRA), and lower solidity traits (SOL2). These works have been possible due to the use of computer vision and machine learning in extraction of complex traits.

Advances in computer vision, machine learning, and high-throughput phenotyping (HTP) technologies, coupled with efficient statistical methods and collaborative research, have opened the way for more research to be carried out in plants as reviewed in Singh et al. (2016, 2018), Atkinson et al. (2019), Ghosal et al. (2019), Parmley et al. (2019), and Singh A. et al. (2021). The use of these technologies has been implemented in the collection of agronomic and yield estimation traits (Riera et al., 2021), detection of abiotic and biotic stress (Naik et al., 2017; Zhang et al., 2017; Nagasubramanian et al., 2018, 2019), and monitoring plant health (Ghosal et al., 2018). However, as shown in Falk et al. (2020a), computer vision and machine learning-based methods are essential to advance the root phenotyping and large-scale studies (Singh et al., 2016, 2018; Singh D. P. et al., 2021). Root phenotyping is classified depending on where it is carried out, i.e., in controlled environments or in the field, destructive or nondestructive, and whether the HTP uses 2-dimensional (2D) or 3-dimensional (3D) to capture the traits of interest (see reviews, Atkinson et al., 2019; Singh A. K. et al., 2021).

Previous methods developed for extracting roots in the field include destructive methods such as “shovelomics” (Trachsel et al., 2011), the use of soil cores (Wasson et al., 2016), and nondestructive methods such as electrical resistance tomography (Srayeddin and Doussan, 2009), electromagnetic inductance (Shanahan et al., 2015), and ground penetrating radar (Liu et al., 2018). Soil opacity is still a limiting factor to access roots in most field experiments. Controlled environmental methods include the use of rhizotrons, which utilize soil (Rellán-Álvarez et al., 2015), nonsoil methods such as hydroponics (Aski et al., 2021), transparent artificial growth media (Ma et al., 2019), and growth pouches (Tan and Nopamornbodi, 1979). The high-throughput nature of acquiring 2D root images from controlled environments necessitated the development of the image analysis software to extract the traits (Atkinson et al., 2019). Commercially available software includes WinRhizo (Regent Instruments, Quebec, Canada). The open-source software available for use includes SmartRoot (Lobet et al., 2011), RootNav (Pound et al., 2013), GiaRoots (Galkovskyi et al., 2012), DART (Le Bot et al., 2010), Ez-Rhizo (Armengaud, 2009), DIRT (Das et al., 2015), ARIA (Pace et al., 2014; Falk et al., 2020a), RhizoVision (Seethepalli et al., 2020), MyRoot (Betegón-Putze et al., 2019), and IJ_Rhizo (Pierret et al., 2013). A combination of the methods above has been used to study the roots of a variety of plants under various conditions. Species of plant roots studied include common bean (Bonser et al., 1996), maize (Hund et al., 2009; Zheng et al., 2020), wheat (Atkinson et al., 2015), pearl millet (Passot et al., 2016), soybean (Falk et al., 2020a), and canola (Gioia et al., 2016). In a recent study, Aski et al. (2021) utilized modified hydroponics to study the RSA phenotypic diversity of the mung bean mini-core collection at the World Vegetable Center (formerly known as Asian Vegetable Development and Research Center [AVRDC]) (Schafleitner et al., 2015). As this software is capable of generating the useful data on multiple phenotypic root traits, these also lend themselves to genetic studies.

Genome-wide association studies (GWAS) is a statistical tool that uses historical recombination events to uncover the significant genotypic variation associated with the phenotypic variation for the trait of interest (Huang and Han, 2014; Tibbs Cortes et al., 2021). GWAS has been extensively used to investigate important agronomic traits such as plant height, days to flower, yield, nutrient content, flood and drought tolerance, and insect and pest resistance in maize (Yang et al., 2014), soybean (Zhang et al., 2015; Fang et al., 2017), common bean (Kamfwa et al., 2015), mung bean (Sandhu and Singh, 2021), and rice (Huang et al., 2010) among others.

The current study was conducted with the objectives to (1) study the diversity of the RSA trait in the Iowa (IA) mung bean panel, (2) contextualize these RSA traits with root-based ideotypes, and (3) conduct GWAS on RSA traits and identify candidate genes for these associations.

## Materials and Methods

### Plant Materials

A total of 376 accessions were used in this study. A total of 372 Plant Introductions (PI) were filtered from the 482 IA mung bean panel (Sandhu and Singh, 2021) using the identity-by-state method in SNPRelate and genetic distance of Nei (Zheng et al., 2012). PIs that were common among the two methods were dropped. The 482 PIs were a part of the over 3,000 mung bean accessions obtained from the United States Department of Agriculture-Germplasm Resources Information Network (USDA-GRIN), in Griffin Georgia that were able to flower and form pods in IA conditions. Three Asian Vegetable mung bean (AVMU) lines, namely, AVMU001, AVMU0201, and AVMU9701, were included as checks, since they are improved cultivars from the WVC, formerly AVRDC (Fernandez and Shanmugasundaram, 1988).

### Experimental Design and Germination Protocol

**FIGURE 1:**
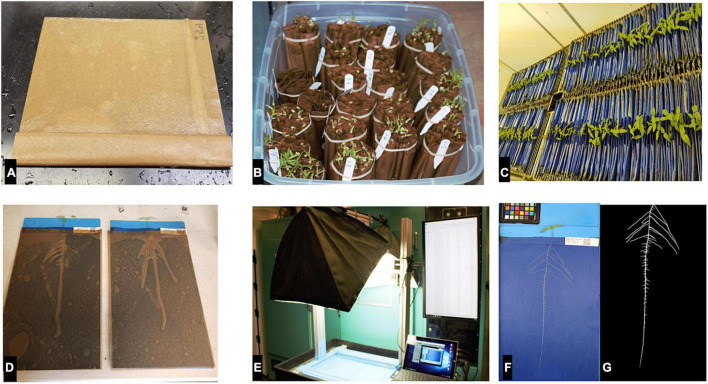
Workflow from seed to phenotyping roots, **(A)** germination roll, **(B)** germination rolls banded by sub-block, **(C)** seedlings in the four blocks inside the growth chamber, **(D)** genotypes pooled out for imaging, **(E)** imaging platform, **(F)** captured root, and **(G)** preprocessed root ready for trait extraction.

This study used a randomized incomplete block design, with each growth chamber serving as a replicate, for a total of eight replications for the experiment. Two growth chambers were used for an increased throughput. Each chamber had four blocks. Each block contained six complete and two incomplete sub-blocks. Each complete sub-block held twelve genotypes, while each incomplete sub-block held eleven genotypes. The genotypes were randomized within each block and sub-blocks. Randomization was generated using the R package blocksdesign (Edmondson and Edmondson, 2021). The procedures described in Falk et al. (2020a) were followed with little modification in the experimental design (Figure 1). First, ten seeds of each genotype were equally spaced near the top (∼1″) of a 9″ × 12″ germination paper. The paper was rolled into germination rolls. All the germination rolls for each sub-block were rubber banded and labeled with a tag. Once all the 376 were planted, water was filled halfway in the rectangular bucket, and the rolls transferred to a Conviron growth chamber (Controlled Environments Ltd., Winnipeg, Canada) set at 25°C for 16 h of light and 20°C for 8 h darkness. The lighting was set to 276–280 μmol/s/m^2^ and constantly monitored using the LI-250A photometer (Li-Cor Biosci-sciences, Lincoln, NE, United States). On the 5th day of germination, a representative sample for each genotype was picked and carefully placed onto the 12″ × 18″ blue germination paper (Anchor Paper, Minneapolis, MN, United States). Labeled bar-coded tags are stapled onto the 1″ folded top of the blue paper. A 12″ × 16″ brown blotting paper (Anchor Paper, Minneapolis, MN, United States) was carefully placed on top of the blue paper. Two full blue papers are clipped together using binder clips and placed in the rack on the plot number in the chamber. Chamber conditions were monitored daily.

### Imaging, Image Processing, and Trait Extraction

A high-throughput imaging station was set up similar to the one reported by Falk et al. (2020a). Images were captured using a Canon T5i digital SLR camera (lens: EF-S 18–55 mm f/3.5–5.6 IS II) (Canon USA, Inc., Melville, NY, United States). The setup allowed for the automated renaming of images captured using the SmartShooter software (Hart, 2021; Figure 1E). The seedlings were imaged on days 12, 15, and 18 without moving roots. Exceptions to moving were on day 12 where some secondary roots did not emerge from the fold and on day 18, when some of the roots of the genotypes were overgrowing the length of the blue paper. The days to image and the spacing were determined by a preliminary study. On the 18th day, the seedlings were cut at the junction between the shoot and camera-visible root section. The root and shoot of each genotype were placed in small brown bags. The wet-cut seedlings were dried in growth chambers set at 34°C/24 h for 2 days, with the light set to 276–280 μmol/s/m^2^ and stored for weighing. Each root and shoot of the genotype were measured using the Ohaus portable weighing balance (Ohaus Corporation, NJ, United States).

More than 9,000 (376 genotypes × 8 reps × 3 time points) images were collected from the whole experiment. The images were first rotated manually to portrait orientation to enable consistent preprocessing. Images with no germinated seed, herein referred to as blank, were excluded from processing. JPEGCrops (2021), an open-source software, was used to auto crop all the images in a batch by cutting off the top part with the labeled bar-coded tag. The images were then converted into black/white images by thresholding (heuristically using red, green, and blue, LAB, or Hue, Saturation, and Value color spaces). This was carried out using the image processing step and followed by the trait extraction step within the improved Automatic Root Image Analysis (ARIA) 2.0 tool (Falk et al., 2020a). Different color spaces were used due to the variations in the images caused by unequal lightning and water spots. The ARIA 2.0 tool runs on Matlab (2020a). Traits extracted in ARIA are shown in the Supplementary Materials (Supplementary Table 1).

### Statistical Analysis

All the analyses were carried out using the R statistical software (R Core Team, 2021). A separate code was written for the extraction of median LRA. Outliers were filtered out using the Tukey’s box plot method (Hoaglin, 2003). A soybean genotype previously included was dropped due to clear visual differences with mung beans. The preprocessing steps above left 8,611 observations represent 375 genotypes for the analysis. Most of the reported analysis is from day 15 data with references and comparisons to days 12 and 18. Day 15 was chosen as a good representation of root growth between day 12 and day 18. A subset of ARIA traits was used for the analysis (Table 1). They were informed by traits used in a similar study by Aski et al. (2021) and traits important for the iRoot categories: topsoil foraging, and steep, cheap, and deep ideotypes described by Falk et al. (2020b). A mixed linear model (Eq. 1) was used to extract the best linear unbiased predictors (BLUPs) for each trait per genotype. All model variables were considered a random effect except chamber, which was a fixed effect. The model was run within the H2Cal function from the inti package (Lozano-Isla, 2021), which utilized the unbalanced data (Cullis et al., 2006; Piepho and Möhring, 2007; Schmidt et al., 2019). Broad sense heritability (H) was calculated using Eq. 2 (Cullis et al., 2006). Pearson correlations were used to draw correlations among the root traits.


(1) $$\begin{array}{c} Y_{ijkl}=\mu +{\text{Chamber}}_{i}+{\left(1|\text{Chamber}:\text{Block}\right)}_{ij}+(1|\text{Chamber}:\\ \;\text{Block}:\mathrm{Sub}-\mathrm{block}){}_{ijk}+(1|\text{Genotype}){}_{l}+e{}_{ijkl} \end{array}$$

**TABLE 1:** Subset of traits of mung bean root architecture extracted from the Automated Root Image Analysis (ARIA) software and used for the analysis, clustering, and iRoot category.

Trait name	Symbol	Unit	Trait description
Total root length	TRL	cm	Cumulative length of all the roots in centimeters
Primary root length	PRL*	cm	Length of the primary root in centimeters
TRLUpper	TRLUpper*	cm	Total root length of the upper one third
Depth	DEP	cm	The maximum vertical distance reached by the root system
Width	WID*	cm	The maximum horizontal width of the whole RSA
Diameter	DIA	cm	Diameter of the primary root
Lateral root branches	LRB	Count	Number of lateral root branches
Network area	NWA	Count	The number of pixels that are connected in the skeletonized image
Convex area	CVA	cm^2^	The area of the convex hull that encloses the entire root image
RhizoArea	RHZO	cm^2^	Length of 2 mm surrounding the TRL
Primary root surface area	PRA	cm^2^	Surface area of the primary root
Volume	VOL	cm^3^	Volume of the primary root
Lateral root angles	LRA*	Angle	Root angles along the extent of all lateral roots
Solidity	SOL*	Ratio	The fraction equal to the network area divided by the convex area
Length distribution	LED	Ratio	TRL_Upper_/TRL_Lower_
Total root length-growth rate	TRL_GR*	cm/day	(TRL_day 15 – TRL_day 12)/3

*Traits used for iRoot ideotypes. RSA, root system architecture.

Where μ is the overall population mean, *Y*_*ijkl*_ is the phenotypic trait, Chamber*_*i*_* is the fixed effect of the *i*th growth chamber (1| Chamber:Block)*_*ij*_* is the random interaction effect between the *i*th chamber and the *j*th block (1| Chamber:Block:Sub-block)*_*ijk*_* is the three way random interaction effect between the *i*th chamber, *j*th block and *k*th sub-block (1| Genotype)_*l*_ is the random effect of the *l*th genotype, and *e*_*ijkl*_ is the random error term following the *N*(0,*q*^2^*e*).


(2) $${\text{H}}_{\text{Cullis}}^{2}=1-{\bar{V}}_{\Delta }^{BLUP}/2\delta_{g}^{2}$$

Where $\delta_{g}^{2}$ is the genotypic variance and ${\bar{V}}_{\Delta }^{BLUP}$is the mean variance of the difference of two genotypic BLUPs for the genotypic effect (Schmidt et al., 2019).

### Root Ideotypes, Phenotypic, and Genotypic Diversity

The iRoots were formed by first ranking the genotypes under each trait, getting the sum of the ranks and then ranking the sums for each category. For topsoil foraging, the genotypes were ranked individually under the TRL_GR, WID, and TRL_Upper_. The sum of the ranks was ranked, and this yielded to the final ranking of each genotype. A similar approach was used for the “steep, cheap, and deep” ideotype using the TRL_GR, steep LRA, and SOL2.

The principal component analysis (PCA) and hierarchical clustering were used in both the phenotypic and genotypic clustering of the genotypes using the Euclidean distance matrix. The base R function hclust with methods “complete” and “prcomp” was used. The package factoextra (Kassambara and Mundt, 2020) was used to determine the optimum number of clusters to be used by comparing 30 different indices. The clusters were related to the country of origin. Heat maps were developed according to the trait performance and iRoot category ranking using the Complex Heatmap package (Gu et al., 2016). Phenotypic and genotypic dendrograms were made using the dendextend (Galili, 2015) and circlize (Gu et al., 2014) packages. The pairwise fixation index (Fst) was calculated between the two genotypic clusters using the function genet.dist (method = “WC84”) within the ade4 package (Dray Stéphane, 2007). Fst is an indication of the amount of differentiation within subpopulations, with low Fst indicating high gene flow (low genetic diversity) (Wright, 1965).

### Genome-Wide Association Analysis

In total, 26,550 SNPs (marker data) were obtained using genotype-by-sequencing and preprocessed earlier by Sandhu and Singh (2021). Sites with >15% missing data and minor allele frequency > 0.01 were filtered out. GWAS was carried out using BLUPs on all the trait data. Associations were conducted using the Trait Analysis by aSSociation, Evolution, and Linkage (TASSEL, Bradbury et al., 2007) software using a linear mixed model (Yu et al., 2006). Both the kinship matrix and PCA were generated in TASSEL controlling for population structure. Bonferroni correction with p-value = 0.05 was used to control for false positives and declare significant associations (Kuo, 2017). Manhattan plots for visualizing the associations were carried out in R using the CMplot library in the rMVP package (Yin et al., 2021). Authors also used a newly developed computational framework, selection of variables with embedded screening (SVEN), a Bayesian based model to run GWAS (Li et al., 2020). The identification of candidate genes was carried out by locating the significant SNP on the sequenced and annotated mung bean genome using the “genome data viewer” tool at the National Center for Biotechnology Information (NCBI; Kang et al., 2014).

## Results

### Descriptive Statistics, Correlation, and Heritabilities

We observed the significant phenotypic variability for root traits. The coefficient of variation (CV) ranged from 2 to 19% and standard deviation (SD) from 0.01 to 628.67 (different units of measurements for traits). Most of the traits had low SD, i.e., <10 except for TRL, VOL, TRL_Upper_, and CVA that had SD < 100, while RHZO had an SD > 500. TRL, TRL_Upper_, CVA, WID, NWA, RHZO, and TRL_GR had 10% < CV < 20%, while the rest of the traits had CV < 10% (Table 2). Day 12 CV ranged from 0 to 22% while for day 18 was 0–28%. Dry matter weight measurements, including shoot dry weight (SDW), root dry weight (RDW), and total dry weight (TDW) had CV 24, 28, and 26%, respectively, at day 18 (Supplementary Table 2).

**TABLE 2:** Descriptive statistics and broad sense heritability of a subset of root traits from day 15 of the Iowa (IA) mung bean genotypes estimated from eight replications.

Trait	Mean	Median	Min	Max	SD	CV (%)	H
TRL	230.22	225.56	159.22	325.48	35.1	15	0.66
PRL	42.72	42.65	36.48	48.06	1.7	4	0.54
LED	2.05	2.04	1.53	2.6	0.19	9	0.51
DIA	0.24	0.24	0.23	0.26	0.01	3	0.31
VOL	261.41	260.76	223.14	316.38	17.76	7	0.29
Surface area	31.42	31.45	28.9	34.76	1.06	3	0.24
TRL_Upper_	150.85	149.35	99.82	216.32	22.63	15	0.64
CVA	412.66	407.67	258.73	567.28	65.28	16	0.66
DEP	37.74	37.7	33.47	40.66	1.16	3	0.53
WID	18.68	18.4	11.69	25.47	2.88	15	0.73
NWA	2.82	2.77	1.98	3.95	0.41	15	0.64
LRB	137.67	137.82	124.39	152.1	4.28	3	0.32
RHZO	4651.19	4588.15	3272.6	6280.68	628.27	14	0.63
SOL2	140.92	140.94	107.82	164.14	9.6	7	0.57
LRA	50.23	50.18	46.64	53.36	1.11	2	0.22
TRL_GR	24.64	23.88	15.76	39.69	4.64	19	0.68

*Full trait descriptions are in Table 1. SD, standard deviation; CV, coefficient of variation; H, broad sense heritability.*

**FIGURE 2:**
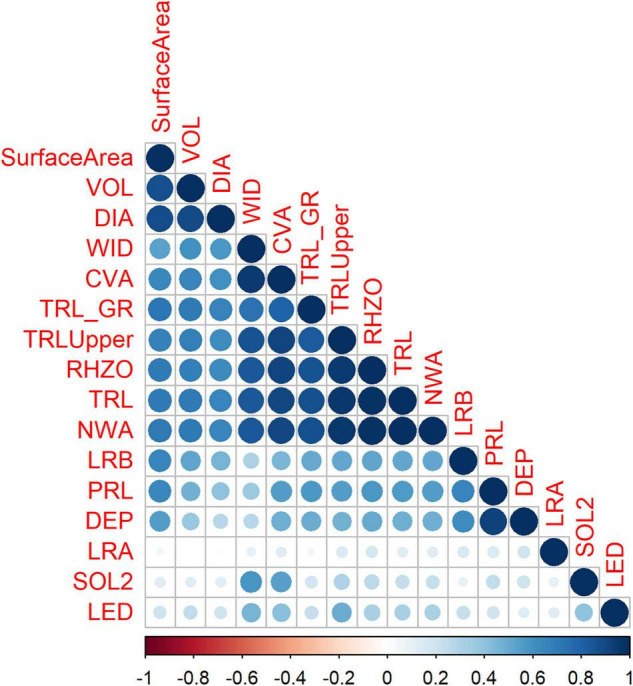
Correlations among the root traits at day 15 using the Iowa (IA) mung bean panel. Experiments were conducted in controlled environment chambers and included eight replications.

The correlation between the root traits varied. TRL_Upper_ was highly correlated with WID, CVA, and TRL_GR. NWA was highly correlated with WID, CVA, TRL_GR, TRL_Upper_, RHIZO, and TRL. LRA had the lowest correlation with the other traits. There was no correlation between LRA and VOL, and DIA (Figure 2). Correlation on day 12 was high. Negative correlations were observed at day 18 with SOL2 being negatively correlated to most traits and LRA negatively correlated with root shoot ratio (RSR) (image not shown).

**FIGURE 3:**
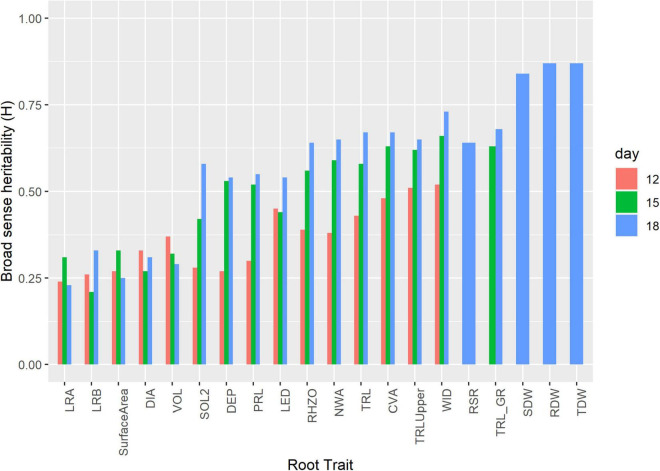
Broad sense heritability (H) of select root related traits at days 12, 15, and 18. Experiments were carried out with the IA mung bean panel with genotypes grown in growth chambers.

Broad sense H ranged from 0.22 to 0.73. LRA and WID had the lowest and highest H at 0.22 and 0.73, respectively. DIA, VOL, surface area, LRB, and LRA had H < 0.5, while TRL, PRL, LED, TRL_Upper_, CVA, DEP, WID, NWA, RHZO, SOL2, and TRL_GR had H > 0.5 (Table 2 and Figure 3). H was high at days 15 and 18 and low on days 12 for most of the traits. Day 12 H ranged from 0.24 to 0.51, with TRL_Upper_ having the highest H. Day 18 H ranged from 0.23 to 0.87, with dry weight traits (i.e., SDW, RDW, and TDW) showing higher levels at 0.84, 0.87, and 087, respectively (Supplementary Table 2).

### Root Ideotypes

**FIGURE 4:**
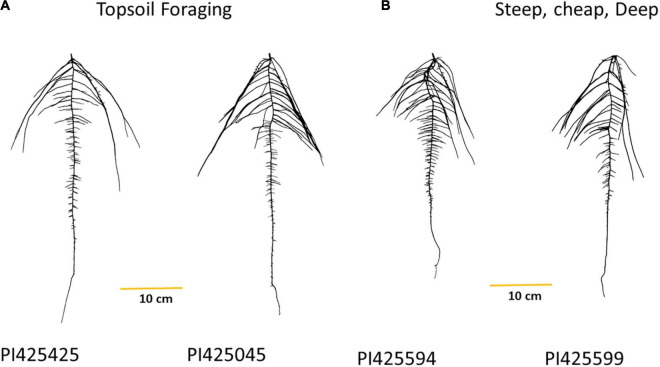
Mung bean iRoot ideotypes, **(A)** top two genotypes in the topsoil foraging and **(B)** top two genotypes in the steep, cheap, and deep after ranking the genotypes in the IA mung bean panel.

We described two root ideotypes, namely, topsoil foraging and “steep, cheap, and deep.” PI425425 (India), PI425045 (Philippines), PI425551 (Korea), PI264686 (Philippines), and PI425085 (Sri Lanka) emerged as top five in the topsoil foraging category. PI425594 (unknown origin), PI425599 (Thailand), PI425610 (Afghanistan), PI425485 (India), and AVMU0201 (Taiwan) were top five in the “steep, cheap, and deep” ideotype (Table 3 and Figure 4). For day 18, the PI425551 (Korea), PI264686 (Philippines), PI426026 (Thailand), PI425085 (Sri Lanka), and PI426042 (Australia) were the top five in the topsoil foraging category. In the “steep, cheap, and deep” ideotype, PI264686 (Philippines), PI425551 (Korea), PI363514 (India), and PI425599 (Thailand) were the top four (Supplementary Table 3). No iRoot categories were created on day 12 since TRL_GR could not be calculated.

**TABLE 3:** Top five genotypes by iRoot rank categories from day 15 image analysis.

Topsoil foraging	Country	“Steep, cheap, and deep”	Country
PI425425	India	PI425594	Unknown origin
PI425045	Philippines	PI425599	Thailand
PI425551	Korea	PI425610	Afghanistan
PI264686	Philippines	PI425485	India
PI425085	Sri Lanka	AVMU0201	Taiwan

### Phenotypic and Genotypic Clusters

**FIGURE 5:**
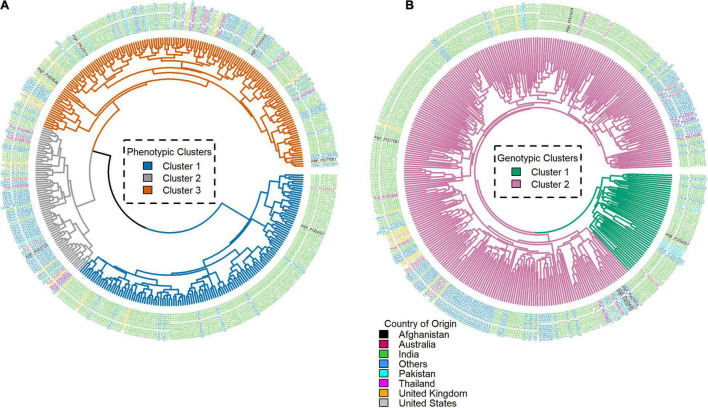
Day 15 phenotypic **(A)** and genotypic **(B)** clusters of the root traits and single-nucleotide polymorphism (SNP) data, respectively, generated using hierarchical clustering of the core traits for all the genotypes. The labels represent the Plant Introductions (PI) and country of origin.

Three distinct phenotypic clusters were observed using the root trait data, while two clusters were observed from the SNP data of the genotypes (Figure 5). Phenotypic clusters 1, 2, and 3 had 69, 163, and 135 genotypes, respectively. Genotypic clusters 1 and 2 had 48 and 319 genotypes, respectively. India had the highest number of genotypes in both genotypic clusters 1 (37) and 2 (197). The United Kingdom had 13 genotypes in genotypic cluster 2, while the rest of the countries had less than 10 genotypes in each cluster. The United Kingdom and United States had no genotypes in genotypic cluster 1 (Supplementary Figure 1). Similarly in the phenotypic clusters 1, 2, and 3, India led with 17, 94, and 123 genotypes. The rest of the countries had less than ten genotypes (Supplementary Table 3). On day 18, there were two phenotypic clusters and two genotypic clusters. Phenotypic clusters 1 and 2 had 250 and 117 genotypes, respectively. Genotypic clusters had similar composition as from day 15. In the phenotypic cluster, India had 132 and 102 genotypes in clusters 1 and 2. The rest had less than 10 genotypes (Supplementary Figure 2 and Supplementary Table 4).

**FIGURE 6:**
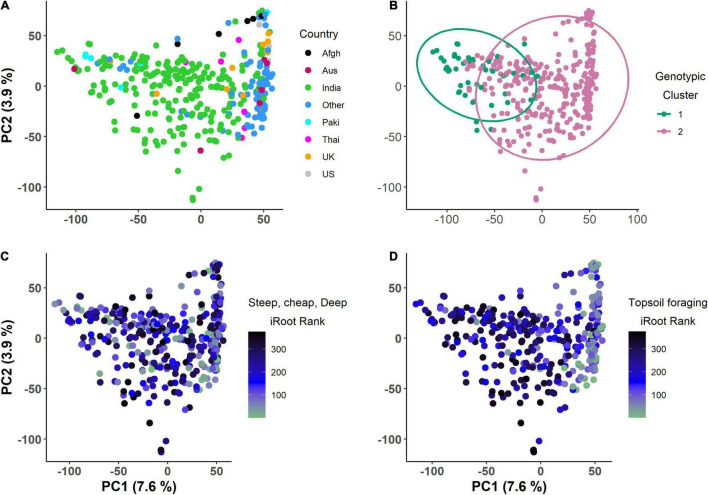
Day 15 principal component analysis of the genotypes for the IA mung bean panel, **(A)** colored by country of origin, **(B)** colored by genotypic clusters, **(C)** color gradient showing ranking in the steep, cheap, and deep iRoot category, and **(D)** color gradient showing ranking in the topsoil foraging iRoot category (the lower the rank, the better the genotype).

**FIGURE 7:**
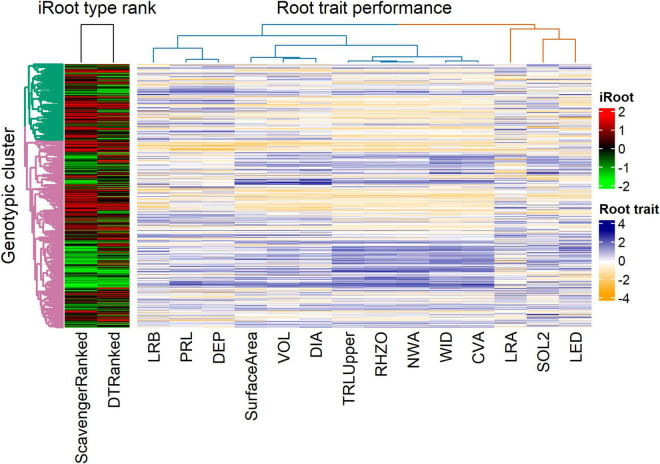
Heat map revealing patterns between the genotypic clusters, iRoot category, and root traits performance for the genotypes in the IA mung bean panel. The dendrogram on the y-axis represents the genotypic clusters (green = 1 and pink = 2). The dendrograms on the top represent the iRoot category and root trait performance.

PC1 and PC2 explained 7.6 and 3.9% of the total genotypic variation in the IA mung bean GWAS panel (Figure 6). The PCs were not able to discern any distinct subpopulations. Superimposition of iRoot ranking on the PCs showed that genotypes from India dominated both in the “steep, cheap, and deep” and topsoil foraging (Figures 6C,D). For day 18, the top genotypes in the “steep, cheap, and deep” category are mostly from India, while in the topsoil foraging, they are mostly from the other countries, Australia, the United Kingdom, and others with few from India (Supplementary Figure 3). The complex heat map showed the patterns and correlations among the genotypic clusters, iRoot type rank, and root trait performance used in clustering (Figure 7). Most of the traits in genotypic cluster 2 had a better ranking in the topsoil foraging, while cluster 1 contained mostly the worst ranked in the same category. Genotypes were evenly distributed in ranking among the genotypic clusters 1 and 2 in the “steep, cheap, and deep” iRoot category. Some of the best genotypes for the traits, including TRL_Upper_, RHZO, NWA, WID, and CVA, were in genotypic cluster 2, while cluster 1 was dominated by low values in the above traits. LRA, SOL2, LED, LRB, PRL, and DEP looked evenly distributed within genotypic clusters 1 and 2 (Figure 7). The pairwise Fst was 0.05.

### Genome-Wide Association Studies and Candidate Genes

**FIGURE 8:**
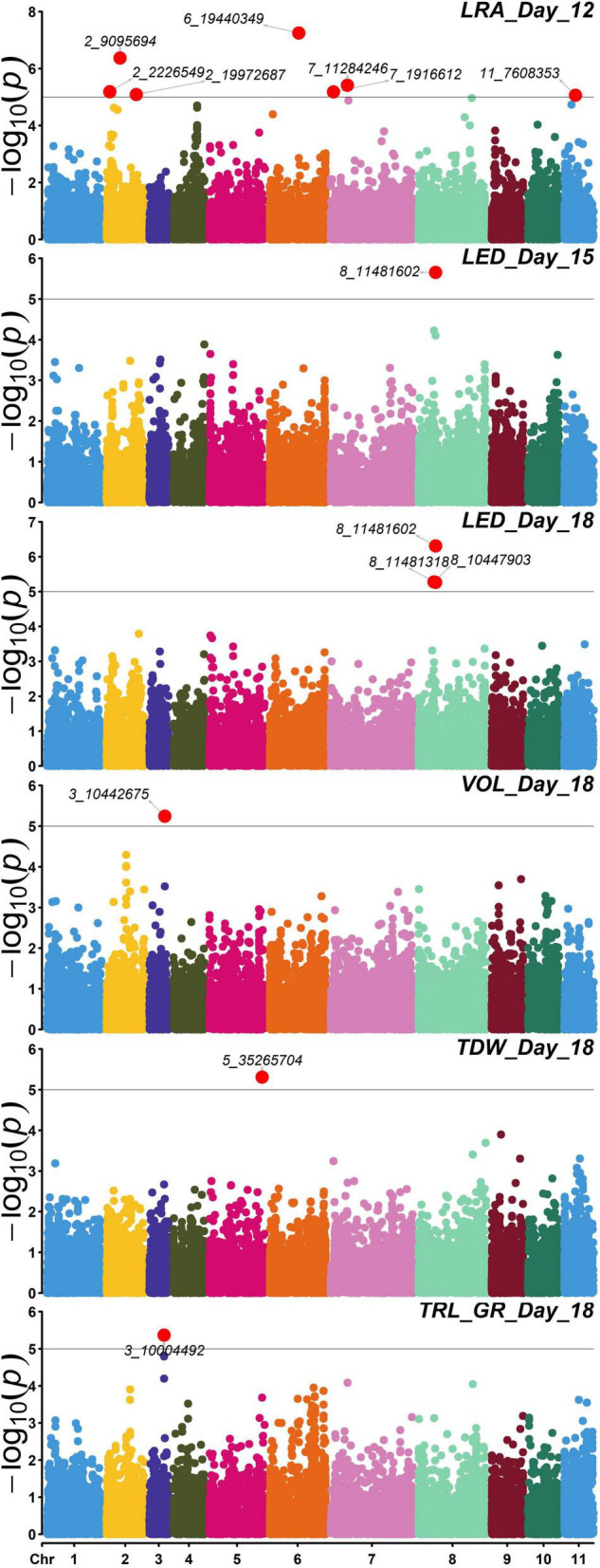
Manhattan plots of –log10(*p*) vs chromosomes of SNP markers associated with the mung bean traits lateral root angles (LRA) (day 12), length distribution (LED) (day 15), and volume (VOL), LED, total dry weight (TDW), total root length-growth rate (TRL_GR) for day 18. The threshold line is the Bonferroni correction at *p* = 0.05 [at –log10(*p*) = 5]. Significant SNPs are highlighted in red and annotated with the marker name. Trait—SNP associations performed using the Trait Analysis by aSSociation, Evolution, and Linkage (TASSEL) software.

Association studies revealed significant SNPs for traits on different days. Day 12 LRA had seven significant SNPs. Day 15 LED had one significant SNP. On day 18, TRL_GR, TDW, and volume each had one significant SNP, while LED had two significant SNPs (Figure 8). Out of the seven SNPs for day 12 LRA, the first three had no mapping on the mung bean genome with no gene ID, genomic context, and gene description. On day 18, significant marker 8_10447903 for LED is an uncharacterized gene. Significant SNPs were found for the same trait LED, for days 15 and 18, marker 8_11481602 and marker 8_10447903, respectively. A summary of the significant SNP associations from the TASSEL software is presented in Table 4.

**TABLE 4:** Significant single-nucleotide polymorphisms (SNPs) for results of association studies for traits across days 12, 15, and 18 as run in the Trait Analysis by aSSociation, Evolution, and Linkage (TASSEL) software.

Day	Trait	Marker	Chr	Pos	p	Add_effect	MarkerR2	Gene ID	Genomic context	Gene description
12	LRA	6_19440349	6	19440349	5.65E-08	–1.53E+00	0.10564	None	None	None
	LRA	2_9095694	2	9095694	4.26E-07	1.44515	0.08585	None	None	None
	LRA	7_11284246	7	11284246	3.86E-06	NaN	0.06085	None	None	None
	LRA	2_2226549	2	2226549	6.50E-06	–1.10E+00	0.06824	LOC106755829	Exon	Lignin-forming anionic peroxidase-like
	LRA	7_1916612	7	1916612	6.55E-06	–1.12E+00	0.06827	LOC106768494	Exon	Beta-galactosidase 3
	LRA	2_19972687	2	19972687	8.13E-06	1.54662	0.07675	LOC106753988	Exon	(–)-Germacrene D synthase-like
	LRA	11_7608353	11	7608353	8.66E-06	1.06558	0.06673	LOC106776541	Exon	Protein FAR1-RELATED SEQUENCE 5
15	LED	8_11481602	8	11481602	2.22E-06	0.09192	0.07531	LOC106772343	Exon	Monodehydroascorbate reductase
18	TRL_GR	3_10004492	3	10004492	4.26E-06	–2.48E+00	0.06752	LOC106757974	Exon	Mannose-1-phosphate guanylyltransferase 1
	LED	8_11481602	8	11481602	4.86E-07	0.1291	0.08614	LOC106772343	Exon	Monodehydroascorbate reductase
	LED	8_10447903	8	10447903	5.27E-06	–1.14E-01	0.07111	LOC106771882	Intron	Uncharacterized LOC106771882
	TDW	5_35265704	5	35265704	4.92E-06	0.00902	0.06543	LOC106760865	Exon	Putative dehydration-responsive element-binding protein 2H (DREB2)

Day 12 SNP markers 2_2226549, 2_19972687, 7_19972687, and 11_7608353 were associated with LRA. Marker 2_2226549 is located within an exon for a gene described as lignin forming anionic peroxidase (LOC106755829). Marker 2_19972687 is located within an exon encoding a gene (-)-germacrene D synthase-like (LOC106753988). Marker 7_19972687 is located within an exon of the beta-galactosidase 3 gene (LOC106768494). Marker 11_7608353 associated with LRA also located within an exon for a gene described as protein FAR1-RELATED SEQUENCE 5 (LOC106776541). The same significant SNP marker 8_11481602 associated with LED from days 15 and 18 was found within the exon of a monodehydroascorbate reductase gene (LOC106772343). Day 18 SNP marker 8_10447903 was found within an intron for an uncharacterized gene but close to the LOC106772343 gene. Day 18 SNP marker 3_10004492 associated with TRL_GR is located within the exon for a gene coding for mannose-1-phosphate guanylyltransferase 1 (LOC106757974). Day 18 SNP maker 5_35265704 associated with TDW is located in the exon for a gene described as putative dehydration-responsive element-binding protein 2H (LOC106760865).

**FIGURE 9:**
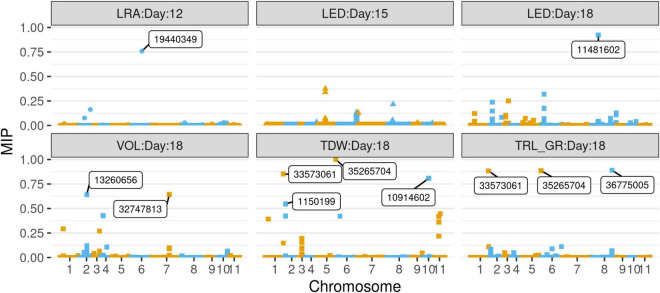
Selection of variables with embedded screening (SVEN) plots of marginal inclusion probability (MIP) vs chromosomes of SNP markers associated with the mung bean traits LRA (day 12), LED (day 15), and LED, VOL, TDW, TRL_GR for day 18. Significant SNPs are boxed with the marker name.

Selection of variables with embedded screening resulted in several significant markers for most of the traits across the 3 days (Figure 9, Supplementary Figures 4–6, and Supplementary Table 5). Two markers for LED (8_44518003) and TDW (5_35265704) from day 18 did overlap with TASSEL results. Marker 8_44518003 is an exon within the gene encoding monodehydroascorbate reductase (LOC106772343), while marker 5_35265704 was found within the gene encoding putative dehydration-responsive element-binding protein 2H (LOC106760865). Day 18 marker for TRL_Upper_ (2_22583526) was found within an exon in the gene encoding coilin (LOC106756657), while marker for DEP (5_23119832) was found in an exon within the gene encoding expansin-A11 (LOC106761944).

## Discussion

Controlled environments have been successfully used to study organisms out of their *in situ* environments (Crop Science Controlled Environment Research Guidelines, 2021). Plants in controlled environments may be exposed to similar conditions as would be in the field to help better achieve the objectives under study (Tibbitts and Langhans, 1993). There have been successful results for measuring various above-ground phenotypes in controlled environments, but below-ground phenotypes pose additional challenges (White et al., 2013). While studies in controlled environments do not imitate what *in situ* root environments look like, they are helpful in *a priori* screening of genotypes to minimize the heavy below-ground phenotyping work required in the field (Lynch and Brown, 2012; Li R. et al., 2015; Ye et al., 2018).

Mung beans are mostly grown on residual moisture after primary crops in most of Southeast Asia (Poehlman and Milton, 1991; Aski et al., 2021). In the Western world, mung beans planted in the summers depend highly on the moisture residue, often following a wet, cold spring. In IA, mung beans are planted around the first week of June, capitalizing on the intense solar radiation for rapid growth (Sandhu and Singh, 2021). This would explain why mung beans, like other legume species, would be ideal with the “steep, cheap, and deep” root ideotypes, to chase the water and the soluble nitrogen before the establishment of root nodules for atmospheric nitrogen fixation. Schneider et al. (2021) showed that steep root angles improved nitrogen uptake *in silico* in maize. Using the OpenSimRoot model, an 11% increase in nitrogen uptake and a 4% increase in plant biomass were predicted at 40 days of growth (Schneider et al., 2021).

Lynch and Brown (2012) showed that common bean genotypes with wide basal root angles were superior in phosphorus (P) acquisition, while the ones with narrow basal root angles were superior in water acquisition during drought conditions. A recent study looked at the P efficiency of mung bean root morphology traits in low and optimum conditions (Reddy et al., 2020), a trait associated with topsoil foraging. They found Indian improved cultivars would be better with regards to P foraging. We identified the top genotypes, including PI425425 (India), PI425045 (Philippines), PI425551 (Korea), PI264686 (Philippines), and PI425085 (Sri Lanka), in the topsoil foraging (Table 3). Our hypothesis is that this represents the improved germplasm developed in India or after migration from India, while some of the lower ranks are still landraces or wild relatives, but this will need to be evaluated further in field conditions. For example, AVMU0201 is from Taiwan, the World Vegetable Center (Brassica, 2014) (formerly AVRDC), which has been breeding mung beans since the 1970s. Accessions PI425045 and PI264686 are from the Philippines, which also hosts a duplicate mung bean collection at the University of the Philippines, Los Banos (Poehlman and Milton, 1991).

We reported a wide variability of the root trait phenotype in the IA mung bean panel during the early stages of development (Table 2). Indian genotypes represented 24, 56, and 89% in the phenotypic clusters 1, 2, and 3 with an overall presence of 67% (Supplementary Figure 4). The high H for traits on days 15 and 18 could be due to better capture of the traits by ARIA unlike day 12 as it might be too early for trait development and differentiation. The high correlation could also be explained by the fact that young plants are utilizing all nutrients for the vegetative growth. These conclusions cannot be assumed to represent the rest of the developmental stages of mung bean plants, prompting the need for further studies. Similar observations were made in soybean (Falk et al., 2020b).

Genetic variability is one of the most important factors in a breeder’s toolbox (Cobb et al., 2019). Indian genotypes were 76 and 67% in the genotypic clusters 1 and 2 with an overall presence of 63% (Supplementary Figure 4). The lack of clear subpopulations as indicated by the PCs shows the homogeneity within the mungbean accessions (Figure 6). Previous studies have shown similar results using simple sequence repeat markers in the Indonesian germplasm (Lestari et al., 2014) and the USDA germplasm (Wang et al., 2018). In other studies, the STRUCTURE (Pritchard et al., 2000) analysis showed between 3 and 6 subpopulations although no clear pattern was seen according to their geographical origins (Lestari et al., 2014; Wang et al., 2018; Sandhu and Singh, 2021). A similar observation, attributed to population admixture, was shown in common bean (Burle et al., 2010). The low Fst of 0.05 shows a high gene flow or low differentiation between the two genotypic clusters. The low Fst in this study confirms similar earlier reports within the USDA collections (Wang et al., 2018) and within the Indonesian germplasm (Lestari et al., 2014). Overall, results indicate a narrow genetic diversity in mung bean (Poehlman and Milton, 1991; Singh D. P. et al., 2021). Early breeders had to opt for mutation breeding to increase the genetic diversity. The narrow genetic base can be explained by the self-pollinated nature, very low cross-pollination frequencies, and poor hybridization of mung bean with other *Vigna* species (Poehlman and Milton, 1991; Singh D. P. et al., 2021). The narrow genetic diversity within the IA panel seems to reflect the fact that most of the accessions were collected on the Indian subcontinent, where mung bean was domesticated (Fuller, 2007). Our results support the idea that, in pulses, the lack of genetic diversity is due in part to the continuous use of a few genotypes as parents in the population development (Kumar et al., 2011). This shows the urgency of breeding efforts to diversify the genetic basis.

Adaptive roots to biotic and abiotic stresses will play a key role in bridging the yield gap in crop plants in the changing climate. A solid understanding of the genetic and environmental factors impacting the RSA will be important to the breeding of stable cultivars (see review, Lynch, 2007; Koevoets et al., 2016). RSA traits associated with response to abiotic stresses, including nutrient deficiency, drought tolerance, salinity, flooding, and temperature, and the underlying candidate genes have previously been studied (see review, Koevoets et al., 2016). Narrow LRA, high LED, and increased LRB were highly correlated to high P accumulation in *Arabidopsis* (Gruber et al., 2013), maize (Zhu et al., 2005), and common bean (Bonser et al., 1996). Auxins and strigolactones are key regulators in root and shoot development. An auxin receptor TRANSPORT INHIBITOR RESPONSE1 (TIR1) was shown to be responsible for the change in LRB as a response to low P levels (Pérez-Torres et al., 2008). Reduced LRB and increased PRL are characteristics of the “steep, cheap, and deep” ideotype, where the plant increases resource allocation to chase water and the mobile N in the deeper soil as evidenced in *Arabidopsis* and maize (Lynch, 2013). The nitrate transporters NRT1.1 and NRT2.1 were identified for the reduced LRB and increased PRL (Linkohr et al., 2002). The extended root system in *Arabidopsis* (Yu et al., 2008), rice, cotton, and poplar (Yu et al., 2013) was attributed to HD-ZIP transcription factor (HDG11) which promotes cell elongation by up-regulating cell wall loosening proteins hence important for drought tolerance.

In the current study, several putative candidate genes were identified for root traits associated with genes involved in the plant growth and development and stress tolerance response (Table 4). Lagrimini et al. (1997) proposed that anionic peroxidases, associated with LOC106755829 (day 12 LRA), play a role in plant host defense using a transformed tobacco (*Nicotiana tabacum* L.) plant. They have also been identified as major enzymes in cell wall lignification and found in large quantities in the xylem tissue (Sasaki et al., 2007). (-)- Germacrene D synthase, associated with LOC106753988 (day 12 LRA), is a member of sesquiterpene synthases family of plant proteins that have the capability of converting a precursor molecule farnesyl diphosphate into many sesquiterpene isoforms (Picaud et al., 2006). (-)- Germacrene D synthase catalyzes the formation (-)- Germacrene D, which is known to have strong effects on insects. Beta-galactosidase 3 is associated with Loci LOC106768494 (day 12 LRA), which has been implicated in adventitious root development *via* transcriptomic studies in mung bean (Li S.-W. et al., 2015). In rice, beta-galactosidase 1 and 2 were found to be highly expressed in the root and shoot seedlings, with less expression in flowers and immature seeds. Beta-galactosidases are important in the breakdown of molecular complexes (carbohydrates, glycolipids, and glycoproteins) that contain galactose (Chantarangsee et al., 2007). Beta-galactosidases would be important in the supply of the required energy from storage reserves during the rapid growth phase. The Far-related sequence (FRS) family, associated with LOC106776541 (day 12 LRA), is conserved among plants. Genes in this family are involved in multiple cellular processes (Lin Ma-FAR1). For example, *Arabidopsis* (*Arabidopsis thaliana*) mutants of *fhy3* were less sensitive to both osmotic and salinity stress while also reducing the ABA-dependent inhibition of seedling root elongation, seedling greening, and germination (Tang et al., 2013; Ma and Li, 2018).

Transcripts of the gene encoding monodehydroascorbate reductase associated with LOC106772343 (day 15 and 18 LED), an antioxidant enzyme, were significantly reduced in the root elongation zone when roots for tall fescue (*Festuca arundinaceaSchreb.cv.* “K-31”) when exposed to water stress (Xu et al., 2015). Water stress is associated with high concentration of reactive oxygen species. A mutation in the *Arabidopsis CYT1* gene encoding mannose-1-phosphate guanylyltransferase 1 associated with LOC106757974 (day 18 TRL_GR) showed deficiency in the cell wall after depletion of GDP mannose. The mutants exhibited radial swelling and accumulation of callose at the root tip. The functional analysis revealed mannose-1-phosphate guanylyltransferase 1 is involved in N-glycosylation during the cellulose synthesis (Lukowitz et al., 2001). An orthologous gene (DREB1A/CBF3 and DREB2A) associated with LOC106760865 (day 18 TDW), in *Arabidopsis*, encodes transcription factors that are involved in activating downstream genes involved in drought and cold stress (Sakuma et al., 2006). In another study, DREB2A proteins were found to increase the stress tolerance by modulating root architecture traits like the lateral root number and root length (Shukla et al., 2006; Agarwal et al., 2010).

Selection of variables with embedded screening loci associated with LOC106756657 (day 18 TRLUpper) and LOC106761944 (day 18 DEP) were associated with the adventitious root development in mung bean like the TASSEL results (Li S.-W. et al., 2015). Coilin is important in the formation of Cajal bodies, which are mostly associated with RNA processes. Kanno et al. (2016) suggest that coilin may be acting in multiple levels fine tuning expression of some genes important for environmental adaptation. Expansins are proteins involved with cell wall loosening and modification, partly mediated by the pH expansion of the cell wall during plant growth (Lee et al., 2001). In rice, Zhiming et al. (2011) identified a gene encoding *EXPA17* that was important for the root hair growth, which requires intensive cell wall modification.

The high H among the dry weight measurements can be used in the selection of parents with the root to shoot ratio (RSR) previously used as a measure of the photosynthetic materials allocations (Figure 3). During a low supply of water, nitrogen, and phosphorus in the soil, more resources are allocated to roots relative to shoots (Xu et al., 2015; Lynch et al., 2021). Within legumes QTLs for fibrous rooting/surface area (Abdel-Haleem et al., 2011), root length (Prince et al., 2015), lateral root number, and root thickness (Manavalan et al., 2015; Prince et al., 2019) in soybean have been mapped. In cowpea, QTLs for basal root angle, root diameter, median width, and width accumulation were reported (Burridge et al., 2017). In pea, root length QTL (Fondevilla et al., 2010) and in common bean basal root angle QTL have been identified. Root length density, root surface area, RDW ratio, and root depth in chickpea have been mapped (Jaganathan et al., 2015). In cereals for, maize and sorghum associations with area, convex hull area, median width, maximum width, width-profile angle, and adjusted depth were identified (Zheng et al., 2020), deep root mass, and the number of deep roots in rice (Courtois et al., 2013) and PRL, RDW in wheat (Sanguineti et al., 2007).

Our study has elucidated the phenotypic and genotypic variability for the root traits in the 375 genotypes in the IA mung bean panel. We identified candidate genotypes that can now be advanced to the greenhouse or field for further testing, especially for the root ideotypes. If their trait response and expression can be confirmed, these can be utilized as parents in the breeding program. Using GWAS, we identified significant markers associated with several RSA traits. Taken together, the ideotypes after field evaluation and significant markers can be utilized as tools for marker-assisted selection and crop improvement in mung bean breeding programs.

## Data Availability Statement

A subset of the dataset and the R scripts used in the analysis can be found in the github account mungbeanpaper, https://github.com/yalek/mungbeanpaper.git. Raw data is provided in the Supplementary Material. Marker dataset can be found at Dryad Data, doi: 10.5061/dryad.wdbrv15mb. Images can be provided upon request.

## Author Contributions

KC and AS conceptualized and designed the experiments. KC performed data collection with the assistance of student workers, did the image preprocessing, data analysis, and wrote the first draft manuscript with feedback from AS and SC on GWAS, and SD and AS on phenotypic analysis. TJ helped with image preprocessing. BG provided feedback on root imaging software. AS provided overall leadership on the project. All authors revised and approved the submitted manuscript.

## Conflict of Interest

The authors declare that the research was conducted in the absence of any commercial or financial relationships that could be construed as a potential conflict of interest.

## Publisher’s Note

All claims expressed in this article are solely those of the authors and do not necessarily represent those of their affiliated organizations, or those of the publisher, the editors and the reviewers. Any product that may be evaluated in this article, or claim that may be made by its manufacturer, is not guaranteed or endorsed by the publisher.
