# Supplementary material for: Dissecting the Root Phenotypic and Genotypic Variability of the Iowa Mung Bean Diversity Panel
Source: Front Plant Sci. 2022 Jan 27;12:808001. doi: 10.3389/fpls.2021.808001 (PMC8828542; doi:10.3389/fpls.2021.808001)
Supplement: Supplementary file 1 [file Table_1.DOCX]

# **Supplementary Materials**


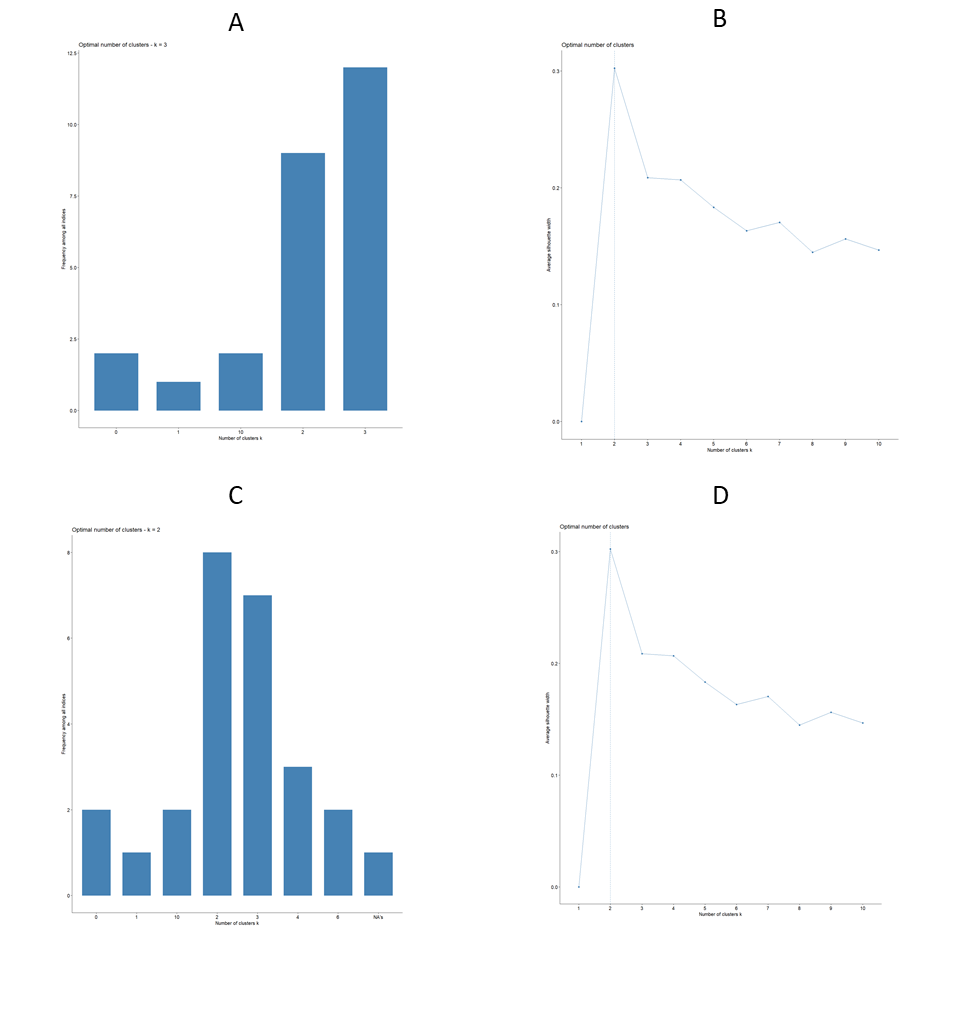


**Figure S1:** Frequency among all indices (y-axis) vs optimal number of clusters (x-axis) as obtained from nbclust. (A) day 15 phenotypic clusters-3, (B) day 15 genotypic clusters-2, (C) day 18 phenotypic clusters -2, (D) day 18 genotypic clusters-2


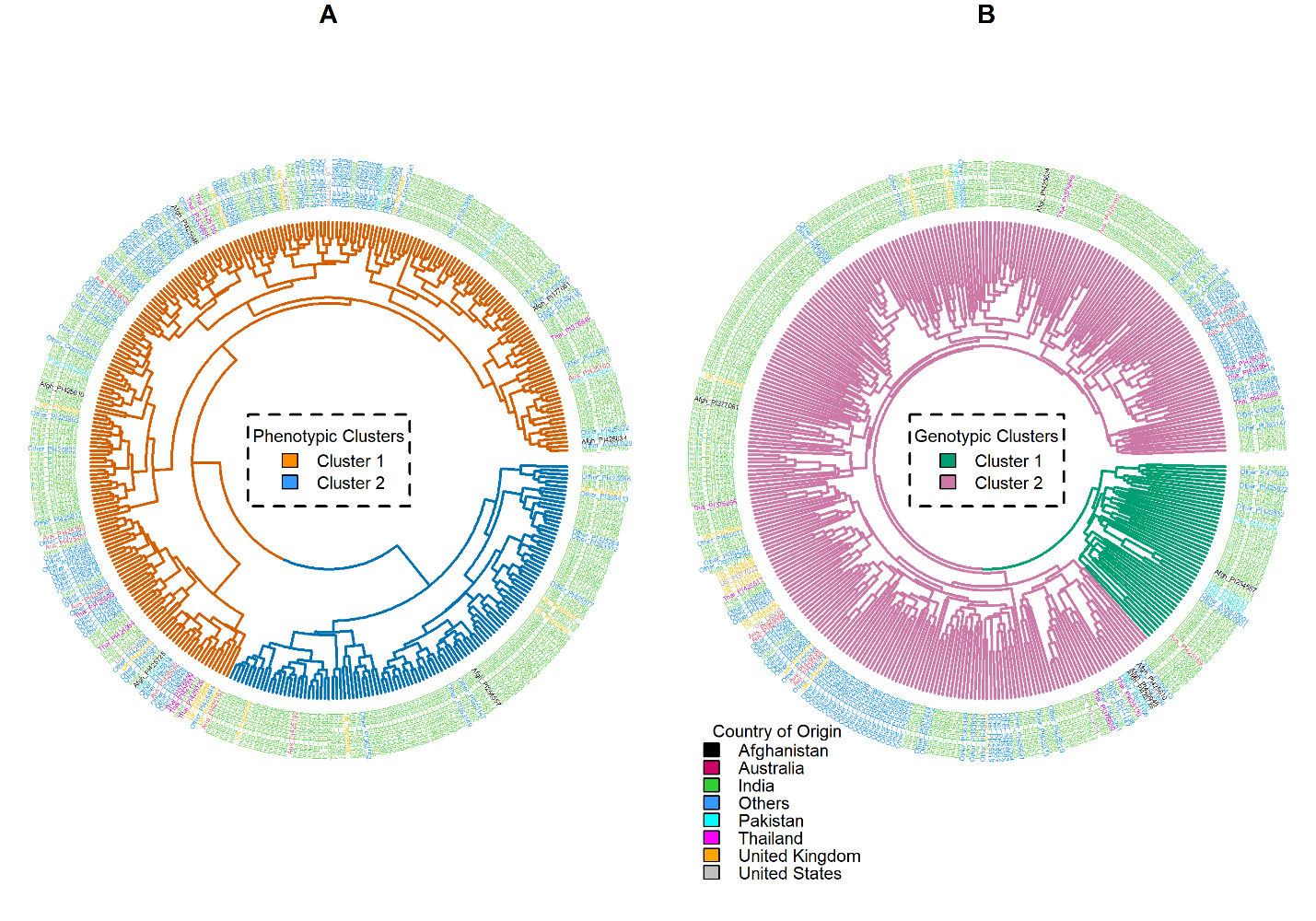


**Figure S2:** Day 18 Phenotypic (A), and genotypic(B) clusters of the root traits and SNP data respectively, generated using hierarchical clustering of the core traits for all the 367 genotypes. The labels represent the PI and country of origin.


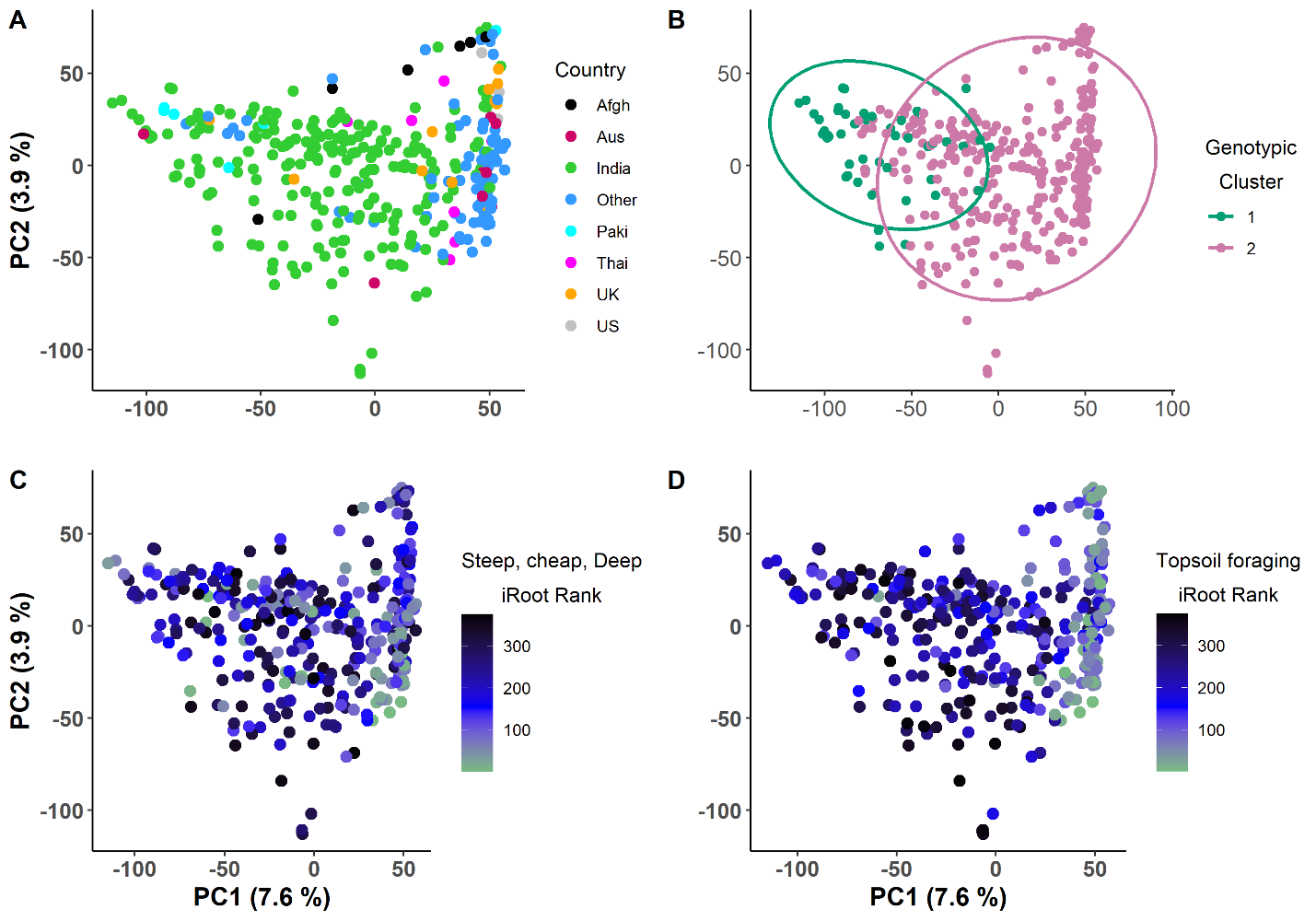


**Figure S3:** Day 18 Principal component analysis of 367 genotypes for the IA mung bean panel, A) colored by country of origin, B) clustered by genotypic clusters, C) color gradient showing ranking in the steep, cheap, and deep iRoot category, D) color gradient showing ranking in the topsoil foraging iRoot category. The lower the rank, the better the genotype.


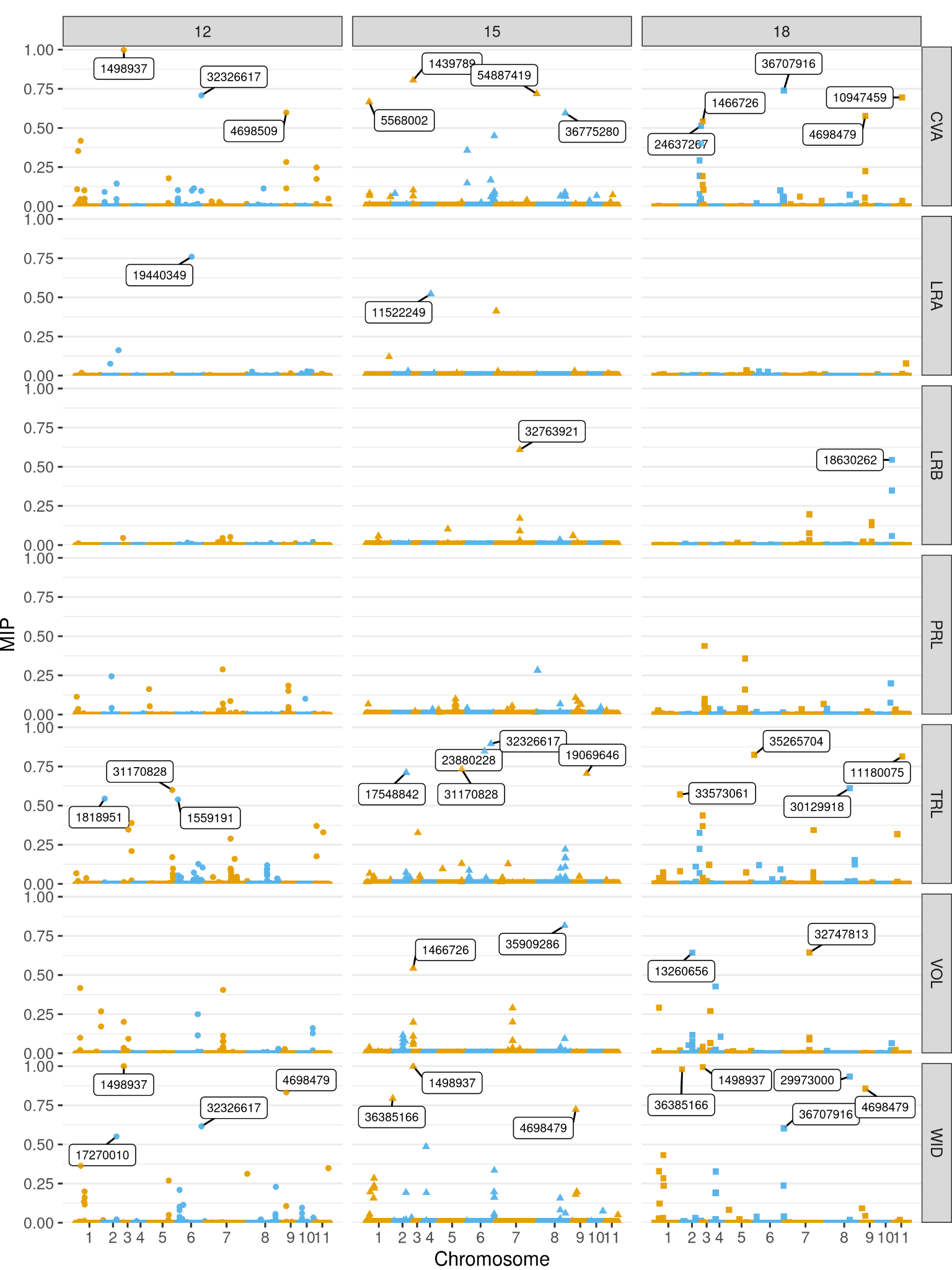


**Figure S4:** SVEN plots of MIP (Marginal Inclusion Probability) vs chromosomes of SNP markers associated with the mung bean traits at day 12, 15 and 18. Significant SNPs are boxed with marker id.


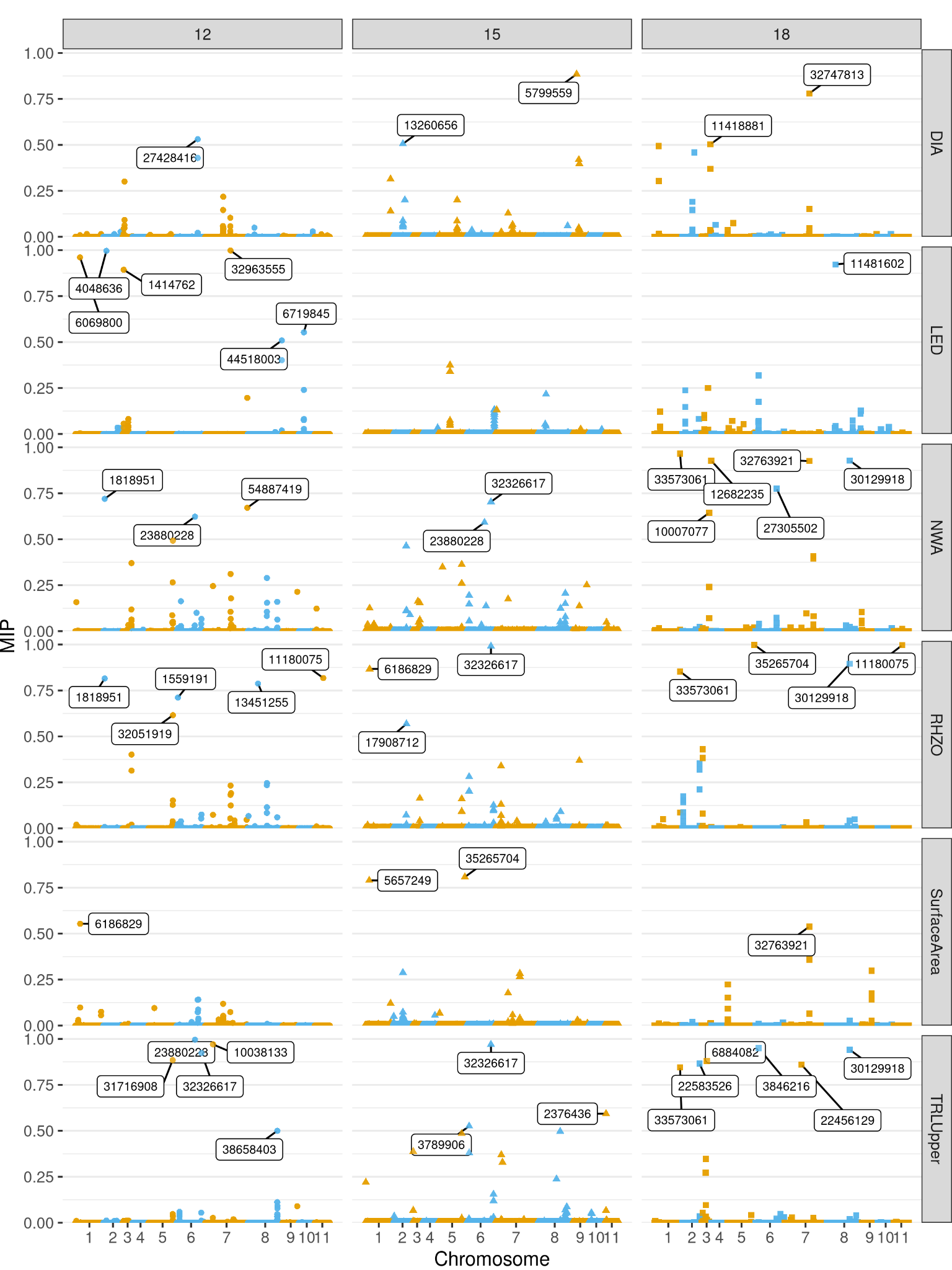


**Figure S5:** SVEN plots of MIP (Marginal Inclusion Probability) vs chromosomes of SNP markers associated with the mung bean traits at day 12, 15 and 18. Significant SNPs are boxed with marker id.


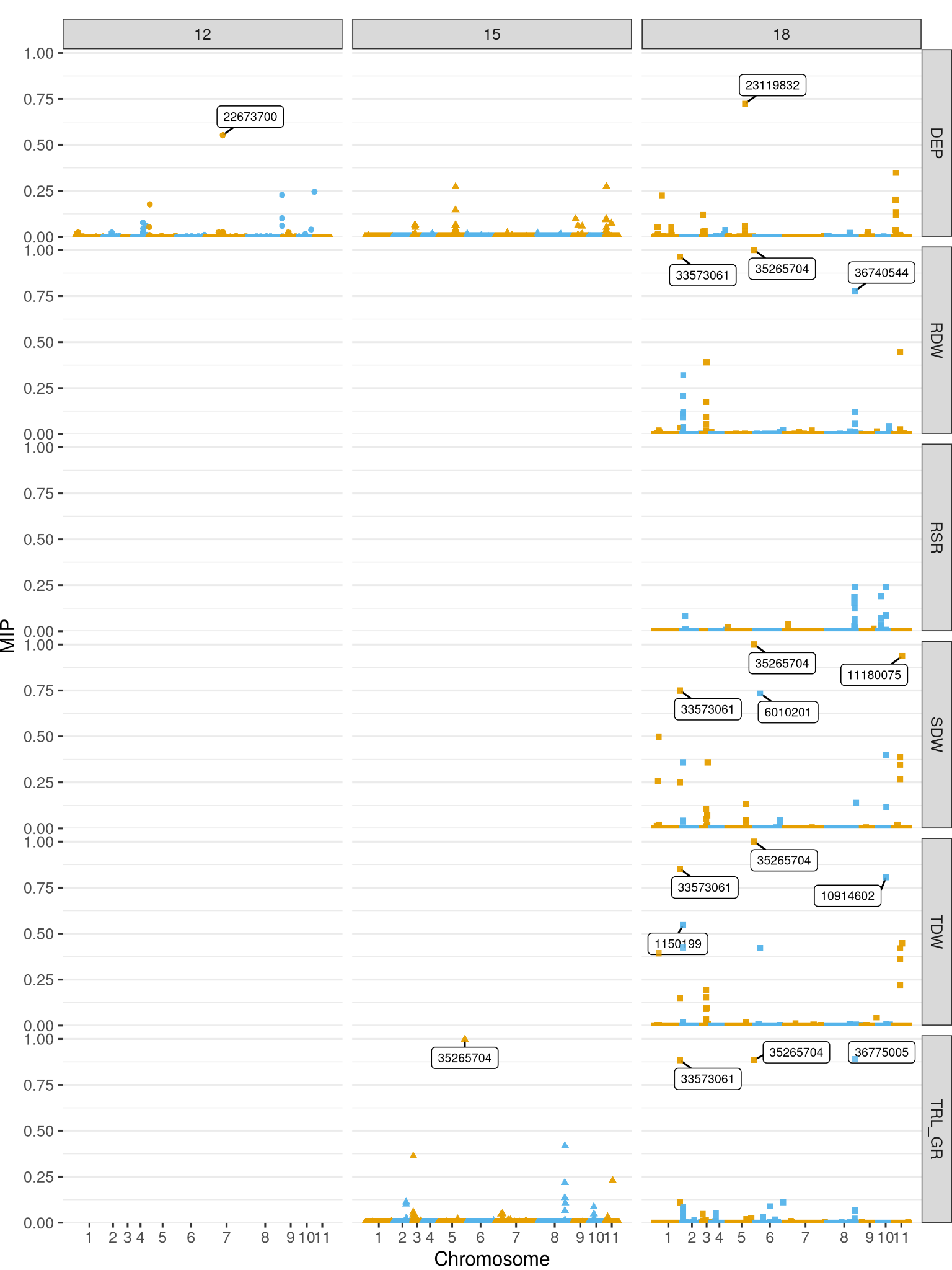


**Figure S6:** SVEN plots of MIP (Marginal Inclusion Probability) vs chromosomes of SNP markers associated with the mung bean traits at day 12, 15 and 18. Significant SNPs are boxed with marker id.

**Table S1:** Root architecture traits derives extracted by the improved Automatic Root Imaging Analysis (ARIA 2.0) software

| **Trait name** | **Symbol** | **Unit** | **Trait description** |
| --- | --- | --- | --- |
| Total root length | TRL | cm | Cumulative length of all the roots in centimeters |
| Primary root length | PRL | cm | Length of the Primary root in centimeters |
| Lateral root length | LRL | cm | Cumulative length of all lateral roots in centimeters |
| Mean lateral root length | MSL | cm | Mean length of all lateral roots in centimeters |
| TRLUpper | TRLUpper | cm | Total root length of the upper one third |
| TRLLower | TRLLower | cm | Total root length of the lower two third |
| Perimeter | PER | cm | Total number of network pixels connected to a background pixel |
| Depth | DEP | cm | The maximum vertical distance reached by the root system |
| Width | WID | cm | The maximum horizontal width of the whole RSA |
| Diameter | DIA | cm | Diameter of the primary root |
| Lateral root branches | LRB | Count | Number of lateral root branches |
| Nodes of lateral roots | NLR | Count | Number of nodes of lateral roots |
| Independent root branches | IRB | Count | Number of independent lateral root branches |
| Lateral root tip | RTA | Count | Number of lateral root tips |
| Median | MED | Count | The median number of roots at all Y-location |
| MaximumR | MAX | Count | The maximum number of roots at all Y-location |
| Maximum number of roots | MNR | Count | The 84th percentile value of the sum of every row |
| Network area | NWA | Count | The number of pixels that are connected in the skeletonized image |
| Convex area | CVA | cm2 | The area of the convex hull that encloses the entire root image |
| RhizoArea | RHZO | cm2 | Length of 2 mm surrounding the TRL |
| TRArea | TRArea | cm2 | Area of the RSA as observed in the 2D projected view |
| Primary root surface area | PRA | cm2 | Surface area of the primary root |
| TRAUpper | TRAUpper | cm2 | Total root area of the upper one third |
| TRALower | TRALower | cm2 | Total root area of the lower two third |
| Volume | VOL | cm3 | Volume of the primary root |
| Lateral root branching angle | LBA | Angle | Lateral root branching angle near the primary root node |
| Lateral root angles | LRA | Angle | Root angles along the extent of all lateral roots |
| Lateral root tip angle | RTA | Angle | Root angle at lateral root tips |
| Width/depth ratio | WDR | Ratio | The ratio of the maximum width to depth |
| Solidity | SOL | Ratio | The fraction equal to the network area divided by the convex area |
| Bushiness | BSH | Ratio | The ratio of the maximum to the median number of roots |
| Length distribution | LED | Ratio | TRLUpper/TRLower |
| LRL by PRL | LSLPL | Ratio | Number of the Lateral root per unit length of the Primary root |
| Center of mass | COM | Ratio | Center of gravity of the root/Depth |
| Center of point | COP | Ratio | Absolute center of the root regardless of root length/Depth |
| Center of mass (Top) | CMT | Ratio | Center of gravity of the top 1/3 of the root (Top)/Depth |
| Center of mass (Mid) | CMM | Ratio | Center of gravity of the middle 1/3 root (Middle)/Depth |
| Center of mass (Bottom) | CMB | Ratio | Center of gravity of the bottom 1/3 root (Bottom)/Depth |
| Center of point (Top) | CPT | Ratio | Absolute center of the root regardless of root length (Top)/Depth |
| Center of point (Mid) | CPM | Ratio | Absolute center of the root regardless of length (Middle)/Depth |
| Center of point (Bottom) | CPB | Ratio | Absolute center of the root regardless of root length (Bottom)/Depth |
| Shoot Dry Weight* | SDW | Grams |  |
| Rood Dry Weight* | RDW | Grams |  |
| Total Dry Weight* | TDW | Grams |  |
| Root to Shoot Ratio* | RSR | Ratio | Root dry weight/shoot dry weight |
| *traits measured manually and calculated | | | |

**Table S2:** Descriptive statistics and heritability of core root traits for days 12 and 18 of the 367 IA mung bean genotypes generated with eight replications.

|  | **Day 12** | | | | | | | **Day 18** | | | | | | |
| --- | --- | --- | --- | --- | --- | --- | --- | --- | --- | --- | --- | --- | --- | --- |
| **Trait** | **Mean** | **Median** | **Min** | **Max** | **SD** | **CV (%)** | **H** | **Mean** | **Median** | **Min** | **Max** | **SD** | **CV(%)** | **H** |
| TRL | 100.36 | 99.67 | 75.38 | 129.04 | 10.4 | 10 | 0.43 | 230.22 | 225.56 | 159.22 | 325.48 | 35.1 | 15 | 0.67 |
| PRL | 34.3 | 34.4 | 31.64 | 36.52 | 0.87 | 3 | 0.3 | 42.72 | 42.65 | 36.48 | 48.06 | 1.7 | 4 | 0.55 |
| LED | 2.29 | 2.28 | 1.7 | 2.92 | 0.22 | 9 | 0.45 | 2.05 | 2.04 | 1.53 | 2.6 | 0.19 | 9 | 0.54 |
| DIA | 0.19 | 0.19 | 0.17 | 0.21 | 0.01 | 4 | 0.33 | 0.24 | 0.24 | 0.23 | 0.26 | 0.01 | 3 | 0.31 |
| VOL | 130.3 | 130.57 | 100.39 | 168.01 | 12.83 | 10 | 0.37 | 261.41 | 260.76 | 223.14 | 316.38 | 17.76 | 7 | 0.29 |
| SurfaceArea | 20.16 | 20.2 | 17.93 | 22.47 | 0.84 | 4 | 0.27 | 31.42 | 31.45 | 28.9 | 34.76 | 1.06 | 3 | 0.25 |
| TRLUpper | 70 | 69 | 46.42 | 96.4 | 10.36 | 15 | 0.51 | 150.85 | 149.35 | 99.82 | 216.32 | 22.63 | 15 | 0.65 |
| CVA | 195.02 | 193.16 | 130.6 | 265.45 | 27.73 | 14 | 0.48 | 412.66 | 407.67 | 258.73 | 567.28 | 65.28 | 16 | 0.67 |
| DEP | 31.49 | 31.54 | 29.18 | 33.52 | 0.67 | 2 | 0.27 | 37.74 | 37.7 | 33.47 | 40.66 | 1.16 | 3 | 0.54 |
| WID | 11.41 | 11.23 | 8.05 | 15.2 | 1.54 | 14 | 0.52 | 18.68 | 18.4 | 11.69 | 25.47 | 2.88 | 15 | 0.73 |
| NWA | 1.22 | 1.21 | 0.95 | 1.54 | 0.11 | 9 | 0.38 | 2.82 | 2.77 | 1.98 | 3.95 | 0.41 | 15 | 0.65 |
| LRB | 99.55 | 99.53 | 90.85 | 108.66 | 2.85 | 3 | 0.26 | 137.67 | 137.82 | 124.39 | 152.1 | 4.28 | 3 | 0.33 |
| RHZO | 2075.47 | 2066.24 | 1615.07 | 2625.15 | 188.6 | 9 | 0.39 | 4651.19 | 4588.15 | 3272.6 | 6280.68 | 628.27 | 14 | 0.64 |
| SOL2 | 143.56 | 143.67 | 130.27 | 157.16 | 4.86 | 3 | 0.28 | 140.92 | 140.94 | 107.82 | 164.14 | 9.6 | 7 | 0.58 |
| LRA | 49.02 | 49.02 | 45.47 | 51.84 | 1.06 | 2 | 0.24 | 50.23 | 50.18 | 46.64 | 53.36 | 1.11 | 2 | 0.23 |
| TRL_GR | NA | NA | NA | NA | NA | NA | NA | 24.64 | 23.88 | 15.76 | 39.69 | 4.64 | 19 | 0.68 |
| SDW | NA | NA | NA | NA | NA | NA | NA | 0.05 | 0.05 | 0.03 | 0.09 | 0.01 | 24 | 0.84 |
| RDW | NA | NA | NA | NA | NA | NA | NA | 0.03 | 0.03 | 0.01 | 0.05 | 0.01 | 28 | 0.87 |
| TDW | NA | NA | NA | NA | NA | NA | NA | 0.08 | 0.08 | 0.04 | 0.14 | 0.02 | 26 | 0.87 |
| RSR | NA | NA | NA | NA | NA | NA | NA | 0.51 | 0.51 | 0.42 | 0.63 | 0.04 | 7 | 0.64 |
| Full trait descriptions are in Table S1.  SD = Standard Deviation, CV = Coefficient of Variation, H = Broad sense heritability | | | | | | | | | | | | | | |

**Table S3:** Top five genotypes by iRoot rank categories for day 18 analysis.

| **Topsoil foraging** | **Country** | **Steep, cheap, Deep** | **Country** |
| --- | --- | --- | --- |
| PI425551 | Korea | AVMU0001 | Taiwan |
| PI264686 | Philippines | PI264686 | Philippines |
| PI426026 | Thailand | PI425551 | Korea |
| PI425085 | Sri Lanka | PI363514 | India |
| PI426042 | Australia | PI425599 | Thailand |

**Table S4:** Genotypic and phenotypic clusters of genotypes grouped by country of origin for days 15 and 18 of the 367 IA mung bean panel.

| **Day 15** | | | **Day 18** | | |
| --- | --- | --- | --- | --- | --- |
| **Country** | **Geno.Cluster** | **n** | **Country** | **Geno.Cluster** | **n** |
| Afgh | 1 | 1 | Afgh | 1 | 1 |
| Afgh | 2 | 5 | Afgh | 2 | 5 |
| Aus | 1 | 1 | Aus | 1 | 1 |
| Aus | 2 | 7 | Aus | 2 | 7 |
| India | 1 | 37 | India | 1 | 37 |
| India | 2 | 197 | India | 2 | 197 |
| Other | 1 | 5 | Other | 1 | 5 |
| Other | 2 | 84 | Other | 2 | 84 |
| Paki | 1 | 4 | Paki | 1 | 4 |
| Paki | 2 | 2 | Paki | 2 | 2 |
| Thai | 2 | 8 | Thai | 2 | 8 |
| UK | 2 | 13 | UK | 2 | 13 |
| US | 2 | 3 | US | 2 | 3 |
|  | **Pheno.Cluster** | **n** |  | **Pheno.Cluster** | **n** |
| Afgh | 1 | 1 | Afgh | 1 | 5 |
| Afgh | 2 | 4 | Afgh | 2 | 1 |
| Afgh | 3 | 1 | Aus | 1 | 7 |
| Aus | 1 | 4 | Aus | 2 | 1 |
| Aus | 2 | 3 | India | 1 | 132 |
| Aus | 3 | 1 | India | 2 | 102 |
| India | 1 | 17 | Other | 1 | 81 |
| India | 2 | 94 | Other | 2 | 8 |
| India | 3 | 123 | Paki | 1 | 6 |
| Other | 1 | 36 | Thai | 1 | 8 |
| Other | 2 | 45 | UK | 1 | 8 |
| Other | 3 | 8 | UK | 2 | 5 |
| Paki | 1 | 1 | US | 1 | 3 |
| Paki | 2 | 5 |  |  |  |
| Thai | 1 | 5 |  |  |  |
| Thai | 2 | 3 |  |  |  |
| UK | 1 | 4 |  |  |  |
| UK | 2 | 7 |  |  |  |
| UK | 3 | 2 |  |  |  |
| US | 1 | 1 |  |  |  |
| US | 2 | 2 |  |  |  |

#

**Table S5:** Significant SNPs for association studies results for traits across days 12, 15, and 18 as SVEN. MIP (Marginal Inclusion Probability)

| **Trait** | **Day** | **Marker** | **Chr** | **Position** | **MIP** | **Gene ID** | **Genomic context** | **Gene description** |
| --- | --- | --- | --- | --- | --- | --- | --- | --- |
| TRL | 12 | 2_1818951 | 2 | 1818951 | 0.54 | LOC106755955 | Exon | GPI inositol-deacylase |
|  |  | 5_31170828 | 5 | 31170828 | 0.60 | LOC106759901 | Exon | myosin-2 |
|  |  | 6_1559191 | 6 | 1559191 | 0.54 | LOC106763224 | Exon | EH domain-containing protein 1-like |
|  | 15 | 2_17548842 | 2 | 17548842 | 0.71 | LOC111240582 | Exon | RING-H2 finger protein ATL14-like |
|  |  | 5_31170828 | 5 | 31170828 | 0.73 | LOC106759901 | Exon | myosin-2 |
|  |  | 6_23880228 | 6 | 23880228 | 0.85 | No gene |  |  |
|  |  | 6_32326617 | 6 | 32326617 | 0.90 | LOC106763719 | Exon | expansin-like B1 |
|  |  | 9_19069646 | 9 | 19069646 | 0.70 | No gene |  |  |
|  | 18 | 1_33573061 | 1 | 33573061 | 0.57 | LOC106769418 | Exon | two pore calcium channel protein 1 |
|  |  | 5_35265704 | 5 | 35265704 | 0.82 | LOC106760865 | Exon | putative dehydration-responsive element-binding protein 2H |
|  |  | 8_30129918 | 8 | 30129918 | 0.61 | No gene |  |  |
|  |  | 11_11180075 | 11 | 11180075 | 0.81 | LOC106777657 | Exon | kinesin-like protein KIN-14B |
| WID | 12 | 2_17270010 | 2 | 17270010 | 0.55 | LOC106755782 | Exon | early nodulin-like protein 2 |
|  |  | 3_1498937 | 3 | 1498937 | 1.00 | LOC106757986 | Exon | serine/threonine-protein kinase Nek4 |
|  |  | 6_32326617 | 6 | 32326617 | 0.62 | LOC106763719 | Exon | expansin-like B1 |
|  |  | 9_4698479 | 9 | 4698479 | 0.83 | LOC106773153 | Exon | proline-, glutamic acid- and leucine-rich protein 1 |
|  | 15 | 1_36385166 | 1 | 36385166 | 0.79 | LOC106762871 | Exon | homeobox-leucine zipper protein ANTHOCYANINLESS 2-like |
|  |  | 3_1498937 | 3 | 1498937 | 1.00 | LOC106757986 | Exon | serine/threonine-protein kinase Nek4 |
|  |  | 9_4698479 | 9 | 4698479 | 0.72 | LOC106773153 | Exon | proline-, glutamic acid- and leucine-rich protein 1 |
|  | 18 | 1_36385166 | 1 | 36385166 | 0.98 | LOC106762871 | Exon | homeobox-leucine zipper protein ANTHOCYANINLESS 2-like |
|  |  | 3_1498937 | 3 | 1498937 | 0.99 | LOC106757986 | Exon | serine/threonine-protein kinase Nek4 |
|  |  | 6_36707916 | 6 | 36707916 | 0.60 | LOC106765097 | Exon | calcium-dependent protein kinase 29 |
|  |  | 8_29973000 | 8 | 29973000 | 0.93 | LOC106772655 | Exon | probable GTP-binding protein OBGM, mitochondrial |
|  |  | 9_4698479 | 9 | 4698479 | 0.86 | LOC106773153 | Exon | proline-, glutamic acid- and leucine-rich protein 1 |
| CVA | 12 | 3_1498937 | 3 | 1498937 | 1.00 | LOC106757986 | Exon | serine/threonine-protein kinase Nek4 |
|  |  | 6_32326617 | 6 | 32326617 | 0.71 | LOC106763719 | Exon | expansin-like B1 |
|  |  | 9_4698509 | 9 | 4698509 | 0.60 | LOC106773153 | Exon | proline-, glutamic acid- and leucine-rich protein 1 |
|  | 15 | 1_5568002 | 1 | 5568002 | 0.67 | LOC106771717 | Exon | arginine--tRNA ligase, cytoplasmic |
|  |  | 3_1439789 | 3 | 1439789 | 0.80 | LOC106757067 | Exon | selenocysteine methyltransferase |
|  |  | 7_54887419 | 7 | 54887419 | 0.72 | LOC106766670 | Exon | U5 small nuclear ribonucleoprotein 40 kDa protein |
|  |  | 8_36775280 | 8 | 36775280 | 0.59 | LOC106770991 | Exon | GABA transporter 1 |
|  | 18 | 2_24637267 | 2 | 24637267 | 0.51 | LOC106756307 | Exon | quinone oxidoreductase |
|  |  | 3_1466726 | 3 | 1466726 | 0.54 | No gene |  |  |
|  |  | 6_36707916 | 6 | 36707916 | 0.74 | LOC106765097 | Exon | calcium-dependent protein kinase 29 |
|  |  | 9_4698479 | 9 | 4698479 | 0.58 | LOC106773153 | Exon | proline-, glutamic acid- and leucine-rich protein 1 |
|  |  | 11_10947459 | 11 | 10947459 | 0.69 | LOC106778060 | Exon | protein ecdysoneless homolog |
| LRB | 15 | 7_32763921 | 7 | 32763921 | 0.61 | No gene |  |  |
|  | 18 | 10_18630262 | 10 | 18630262 | 0.54 | No gene |  |  |
| VOL | 15 | 3_1466726 | 3 | 1466726 | 0.54 | Intergenic |  |  |
|  |  | 8_35909286 | 8 | 35909286 | 0.82 | LOC106770592 | Exon | polycomb group protein EMBRYONIC FLOWER 2-like |
|  | 18 | 2_13260656 | 2 | 13260656 | 0.64 | LOC106756178 | Exon | AP-5 complex subunit zeta-1 |
|  |  | 7_32747813 | 7 | 32747813 | 0.64 | No gene |  |  |
| LRA | 12 | 6_19440349 | 6 | 19440349 | 0.76 | No gene |  |  |
|  | 15 | 4_11522249 | 4 | 11522249 | 0.52 | LOC106759443 | Exon | KH domain-containing protein At2g38610 |
| SOL2 | 12 | 6_36832986 | 6 | 36832986 | 0.51 | No gene |  |  |
|  |  | 8_33839719 | 8 | 33839719 | 0.79 | LOC106771933 | Exon | protein HLB1 |
|  | 15 | 8_33839719 | 8 | 33839719 | 0.84 | LOC106771933 | Exon | protein HLB1 |
| LED | 12 | 1_6069800 | 1 | 6069800 | 0.96 | LOC106772866 | Exon | auxin-responsive protein IAA27*** |
|  |  | 2_4048636 | 2 | 4048636 | 1.00 | LOC106754586 | Exon | FT-interacting protein 1 |
|  |  | 3_1414762 | 3 | 1414762 | 0.89 | LOC106757399 | Exon | non-specific lipid-transfer protein-like protein At2g13820 |
|  |  | 7_32963555 | 7 | 32963555 | 1.00 | LOC106768148 | Exon | guanosine nucleotide diphosphate dissociation inhibitor At5g09550 |
|  |  | 8_44518003 | 8 | 44518003 | 0.51 | LOC106770925 | Exon | WAT1-related protein At4g01440 |
|  |  | 10_6719845 | 10 | 6719845 | 0.55 | LOC106775137 | Exon | V-type proton ATPase subunit e1 |
|  | 18 | 8_11481602 | 8 | 11481602 | 0.92 | LOC106772343 | Exon | monodehydroascorbate reductase |
| RHZO | 12 | 2_1818951 | 2 | 1818951 | 0.82 | LOC106755955 | Exon | GPI inositol-deacylase |
|  |  | 5_32051919 | 5 | 32051919 | 0.62 | LOC106761497 | Exon | inorganic pyrophosphatase 2 |
|  |  | 6_1559191 | 6 | 1559191 | 0.71 | LOC106763224 | Exon | EH domain-containing protein 1-like |
|  |  | 8_13451255 | 8 | 13451255 | 0.79 | LOC106771642 | Exon | uridylate kinase |
|  |  | 11_11180075 | 11 | 11180075 | 0.82 | LOC106777657 | Exon | kinesin-like protein KIN-14B |
|  | 15 | 1_6186829 | 1 | 6186829 | 0.87 | LOC106769542 | Exon | AUGMIN subunit 1 |
|  |  | 2_17908712 | 2 | 17908712 | 0.57 | LOC106756254 | Exon | protein IQ-DOMAIN 1 |
|  |  | 6_32326617 | 6 | 32326617 | 0.99 | LOC106763719 | Exon | expansin-like B1 |
|  | 18 | 1_33573061 | 1 | 33573061 | 0.85 | LOC106769418 | Exon | two pore calcium channel protein 1 |
|  |  | 5_35265704 | 5 | 35265704 | 1.00 | LOC106760865 | Exon | putative dehydration-responsive element-binding protein 2H |
|  |  | 8_30129918 | 8 | 30129918 | 0.90 | No gene |  |  |
|  |  | 11_11180075 | 11 | 11180075 | 1.00 | LOC106777657 | Exon | kinesin-like protein KIN-14B |
| TRLUPPER | 12 | 5_31716908 | 5 | 31716908 | 0.88 | LOC106761702 | Exon | 1-aminocyclopropane-1-carboxylate oxidase 1 |
|  |  | 6_23880228 | 6 | 23880228 | 1.00 | No gene |  |  |
|  |  | 6_32326617 | 6 | 32326617 | 0.92 | LOC106763719 | Exon | expansin-like B1 |
|  |  | 7_10038133 | 7 | 10038133 | 0.97 | LOC106768448 | Exon | serine/threonine-protein phosphatase PP2A-2 catalytic subunit |
|  |  | 8_38658403 | 8 | 38658403 | 0.50 | LOC106772465 | Exon | cleavage stimulating factor 64 |
|  | 15 | 6_3789906 | 6 | 3789906 | 0.53 | LOC106765145 | Exon | CSC1-like protein At3g21620 |
|  |  | 6_32326617 | 6 | 32326617 | 0.97 | LOC106763719 | Exon | expansin-like B1 |
|  |  | 11_2376436 | 11 | 2376436 | 0.59 | LOC106777953 | Exon | probable LRR receptor-like serine/threonine-protein kinase At1g12460 |
|  | 18 | 1_33573061 | 1 | 33573061 | 0.85 | LOC106769418 | Exon | two pore calcium channel protein 1 |
|  |  | 2_22583526 | 2 | 22583526 | 0.87 | LOC106756657 | Exon | coilin |
|  |  | 3_6884082 | 3 | 6884082 | 0.88 | LOC106756952 | Exon | pentatricopeptide repeat-containing protein At5g06540 |
|  |  | 6_3846216 | 6 | 3846216 | 0.95 | LOC106763274 | Exon | UPF0481 protein At3g47200-like |
|  |  | 7_22456129 | 7 | 22456129 | 0.86 | LOC106768681 | Exon | DNA topoisomerase 1 |
|  |  | 8_30129918 | 8 | 30129918 | 0.94 | No gene |  |  |
| NWA | 12 | 2_1818951 | 2 | 1818951 | 0.72 | LOC106755955 | Exon | GPI inositol-deacylase |
|  |  | 6_23880228 | 6 | 23880228 | 0.62 | No gene |  |  |
|  |  | 7_54887419 | 7 | 54887419 | 0.67 | LOC106766670 | Exon | U5 small nuclear ribonucleoprotein 40 kDa protein |
|  | 15 | 6_23880228 | 6 | 23880228 | 0.59 | No gene |  |  |
|  |  | 6_32326617 | 6 | 32326617 | 0.70 | LOC106763719 | Exon | expansin-like B1 |
|  | 18 | 1_33573061 | 1 | 33573061 | 0.97 | LOC106769418 | Exon | two pore calcium channel protein 1 |
|  |  | 3_10007077 | 3 | 10007077 | 0.64 | LOC106757860 | Exon | delta-1-pyrroline-5-carboxylate synthase |
|  |  | 3_12682235 | 3 | 12682235 | 0.93 | No gene |  |  |
|  |  | 6_27305502 | 6 | 27305502 | 0.78 | LOC106763646 | Exon | amino acid permease 3-like |
|  |  | 7_32763921 | 7 | 32763921 | 0.93 | No gene |  |  |
|  |  | 8_30129918 | 8 | 30129918 | 0.93 | No gene |  |  |
| SurfaceArea | 12 | 1_6186829 | 1 | 6186829 | 0.55 | LOC106769542 | Exon | AUGMIN subunit 1 |
|  | 15 | 1_5657249 | 1 | 5657249 | 0.79 | LOC106767448 | Exon | cytochrome c oxidase copper chaperone 1 |
|  |  | 5_35265704 | 5 | 35265704 | 0.81 | LOC106760865 | Exon | putative dehydration-responsive element-binding protein 2H |
|  | 18 | 7_32763921 | 7 | 32763921 | 0.54 | No gene |  |  |
| DIA | 12 | 6_27428416 | 6 | 27428416 | 0.53 | LOC106763050 | Exon | DNA topoisomerase 2-binding protein 1-A |
|  | 15 | 2_13260656 | 2 | 13260656 | 0.51 | LOC106756178 | Exon | AP-5 complex subunit zeta-1 |
|  |  | 9_5799559 | 9 | 5799559 | 0.88 | LOC106774316 | Exon | protein REVEILLE 5-like |
|  | 18 | 3_11418881 | 3 | 11418881 | 0.50 | No gene |  |  |
|  |  | 7_32747813 | 7 | 32747813 | 0.78 | No gene |  |  |
| DEP | 12 | 7_22673700 | 7 | 22673700 | 0.55 | No gene |  |  |
|  | 18 | 5_23119832 | 5 | 23119832 | 0.72 | LOC106761944 | Exon | expansin-A11 |
| TRL_GR | 15 | 5_35265704 | 5 | 35265704 | 1.00 | LOC106760865 | Exon | putative dehydration-responsive element-binding protein 2H |
|  | 18 | 1_33573061 | 1 | 33573061 | 0.88 | LOC106769418 | Exon | two pore calcium channel protein 1 |
|  |  | 5_35265704 | 5 | 35265704 | 0.89 | LOC106760865 | Exon | putative dehydration-responsive element-binding protein 2H |
|  |  | 8_36775005 | 8 | 36775005 | 0.89 | LOC106770991 | Exon | GABA transporter 1 |
| SDW | 18 | 1_33573061 | 1 | 33573061 | 0.75 | LOC106769418 | Exon | two pore calcium channel protein 1 |
|  |  | 5_35265704 | 5 | 35265704 | 0.99 | LOC106760865 | Exon | putative dehydration-responsive element-binding protein 2H |
|  |  | 6_6010201 | 6 | 6010201 | 0.73 | LOC106764084 | Exon | cucumisin-like |
|  |  | 11_11180075 | 11 | 11180075 | 0.94 | LOC106777657 | Exon | kinesin-like protein KIN-14B |
| RDW | 18 | 1_33573061 | 1 | 33573061 | 0.97 | LOC106769418 | Exon | two pore calcium channel protein 1 |
|  |  | 5_35265704 | 5 | 35265704 | 0.99 | LOC106760865 | Exon | putative dehydration-responsive element-binding protein 2H |
|  |  | 8_36740544 | 8 | 36740544 | 0.78 | LOC106772576 | Exon | uncharacterized LOC106772576 |
| TDW | 18 | 1_33573061 | 1 | 33573061 | 0.85 | LOC106769418 | Exon | two pore calcium channel protein 1 |
|  |  | 2_1150199 | 2 | 1150199 | 0.55 | LOC106779521 | Exon | luc7-like protein 3 |
|  |  | 5_35265704 | 5 | 35265704 | 0.99 | LOC106760865 | Exon | putative dehydration-responsive element-binding protein 2H |
|  |  | 10_10914602 | 10 | 10914602 | 0.81 | LOC106775424 | Exon | uncharacterized LOC106775424 |

# 
